# Structural optimization and evaluation of novel 2-pyrrolidone-fused (2-oxoindolin-3-ylidene)methylpyrrole derivatives as potential VEGFR-2/PDGFRβ inhibitors

**DOI:** 10.1186/s13065-017-0301-5

**Published:** 2017-08-01

**Authors:** Ting-Hsuan Yang, Chun-I Lee, Wen-Hsin Huang, An-Rong Lee

**Affiliations:** 1 Graduate Institute of Medical Sciences, National Defense Medical Center, No. 161, Section 6, Mingchuan East Road, Taipei, 11490 Taiwan; 2 School of Pharmacy, National Defense Medical Center, No. 161, Section 6, Mingchuan East Road, Taipei, 11490 Taiwan

**Keywords:** Multi-target kinase inhibitor, VEGFR-2 inhibitor, PDGFRβ inhibitor angiogenesis, (2-oxoindolin-3-ylidene)methylpyrrole, Hydrogen-bond-donating

## Abstract

**Background:**

Tumor angiogenesis, essential for tumor growth and metastasis, is tightly regulated by VEGF/VEGFR and PDGF/PDGFR pathways, and therefore blocking those pathways is a promising therapeutic target. Compared to sunitinib, the C(5)-Br derivative of 2-pyrrolidone-fused (2-oxoindolin-3-ylidene)methylpyrrole has significantly greater in vitro activities against VEGFR-2, PDGFRβ, and tube formation.

**Results and discussion:**

The objective of this study was to perform further structural optimization, which revealed certain new products with even more potent anti-tumor activities, both cellularly and enzymatically. Of these, **15** revealed ten- and eightfold stronger potencies against VEGFR-2 and PDGFRβ than sunitinib, respectively, and showed selectivity against HCT116 with a favorable selective index (SI > 4.27). The molecular docking results displayed that the ligand–protein binding affinity to VEGFR-2 could be enhanced by introducing a hydrogen-bond-donating (HBD) substituent at C(5) of (2-oxoindolin-3-ylidene)methylpyrrole such as **14** (C(5)-OH) and **15** (C(5)-SH).

**Conclusions:**

Among newly synthetic compounds, **7** and **13**–**15** exhibited significant inhibitory activities against VEGFR-2 and PDGFRβ. Of these, the experimental results suggest that **15** might be a promising anti-proliferative agent.

**Graphical abstract:**
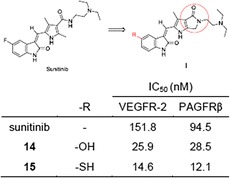
IC_50_ comparison of sunitinib, **14**, and **15** against VEGFR-2 and PDGFRβ.

**Electronic supplementary material:**

The online version of this article (doi:10.1186/s13065-017-0301-5) contains supplementary material, which is available to authorized users.

## Introduction

Angiogenesis is a highly ordered process in which new capillaries are formed from pre-existing vessels in physiological conditions such as reproductive angiogenesis, pregnancy, and wound healing. Angiogenesis is up-regulated in many diseases, including rheumatoid arthritis and especially tumor angiogenesis, which is critical for tumor growth and metastasis [1, 2]. New blood vessels are required for tumor tissues, when beyond 2 mm^3^, to provide oxygen, nutrients, and paths for metastasis, and to remove metabolic wastes [3]. In the absence of vascular support, tumor tissues would become necrotic or apoptotic [4, 5]. Thus, anti-angiogenesis could be an effective therapeutic treatment for cancer.

Pro-angiogenic growth factors secreted by tumor cells, such as angiopoietin-2, epidermal growth factors (EGFs), fibroblast growth factors (FGFs), vascular endothelial growth factors (VEGFs), and platelet-derived growth factors (PDGFs) can stimulate angiogenesis around tumor tissue [6]. Among them, VEGFs, PDGFs, and their receptor tyrosine kinases (RTKs) are the keys of tumor angiogenesis signal transduction [7]. Specific binding of VEGFs and PDGFs to their RTKs triggers downstream signal pathways that induce proliferation, migration, and cell survival of endothelial cells, fibroblast, and vascular smooth muscle cells [8–11]. Therefore, targeting both VEGF and PDGF signal pathways is a promising approach for anti-angiogenesis drug development [9, 10, 12, 13]. Many small-molecule anti-angiogenesis agents targeting VEGFRs and PDGFRs have been developed and approved for clinical use. Of these, sunitinib, an orally bioavailable indolinone-based RTK inhibitor, inhibits angiogenesis by targeting VEGFR-2 and PDGFRβ, and therefore triggers cancer cell apoptosis. The USFDA has approved the use of sunitinib for treating advanced renal cell carcinoma (RCC), gastrointestinal stromal tumors (GISTs) and pancreatic neuroendocrine tumors (pNETs) [7, 14].

Jun et al. showed that the VEGFR-2 and PDGFRβ inhibitory activity of sunitinib was not as potent as those of some novel bicyclic *N*-substituted pyrrolo-fused six-, seven-, and eight-membered-heterocycle derivatives, which are conformation-modified sunitinib analogs. The optimized fused-ring sizes of the products were found to be six and seven. The most potent analog was famitinib, a C(5)-F 2-piperidinone-fused (2-oxoindolin-3-ylidene) methylpyrrole [15]. Famitinib is a tyrosine kinase inhibitor agent targeting at c-Kit, VEGFR-2, PDGFR, VEGFR-3, Flt1, and Flt3. In Phase IIb study, compared to placebo, famitinib showed significantly improved progression free survival (PFS) in patients with advanced colorectal cancer while its toxicity was manageable [16–18].

**Fig. 1:**
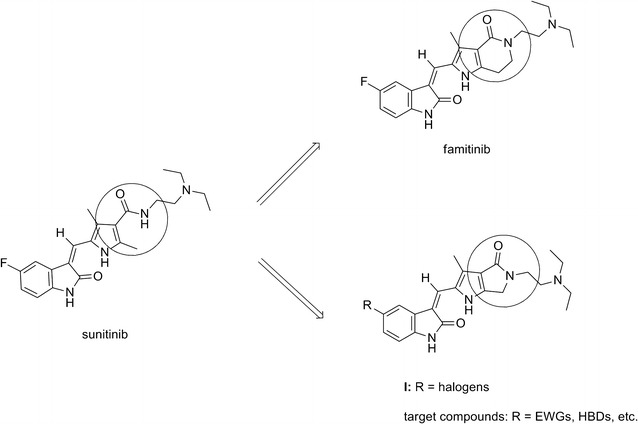
Drug design of target compounds

Given the effectiveness of famitinib, our previous study successfully synthesized a series of novel five-membered-heterocycle derivatives of 2-pyrrolidone fused (2-oxoindolin-3-ylidene)methylpyrrole **I** (Fig. 1) [19]. In contrast to famitinib, our synthetic compounds possess a more rigid conformation than sunitinib and demonstrated superior inhibitory activity of VEGFR-2 and PDGFRβ to sunitinib. Among them, C(5)-Br 2-pyrrolidone-fused (2-oxoindolin-3-ylidene)methylpyrrole showed that potency against VEGFR-2 was fivefold higher in comparison to sunitinib [19].

Structure–activity relationships (SARs) of (2-oxoindolin-3-ylidene)methylpyrrole have been comprehensively investigated in previous works [20–26]. The oxindole scaffold, capable to provide two hydrogen bonds, is critical for the binding of (2-oxoindolin-3-ylidene)methylpyrroles to the ATP-binding site of the kinases, such as VEGFRs [26–28]. The C(5) position of (2-oxoindolin-3-ylidene)methylpyrroles is also considered one of most effective positions for interaction with the ATP-binding site [20–25]. The significant VEGFRs and PDGFRs inhibitory activity of C(5)-halogen substituted 2-pyrrolidone-fused (2-oxoindolin-3-ylidene)methylpyrroles demonstrated in our previous report was at least partly due to increased interaction between the synthetic compounds and the active sites of the receptors [19]. However, it remains unclear whether 2-pyrrolidone-fused (2-oxoindolin-3-ylidene)methylpyrroles with C(5) substituents other than C(5)-halogens, such as groups producing electronic effects by induction or conjugation, are still better VEGFRs and PDGFRs inhibitors. For an improved understanding of the SARs of 2-pyrrolidone-fused (2-oxoindolin-3-ylidene)methylpyrrole with C(5) substituent replacement and in the hope of obtaining novel compounds with more potent anti-proliferative activity and lower toxicity, this study synthesized a series of 2-pyrrolidone-fused (2-oxoindolin-3-ylidene)methylpyrrole with various C(5)-substituents to alter physical and chemical properties for the purpose of ameliorating anti-tumor activity. These experiments revealed several new compounds with favorable selective indexes and potent activities. The most promising of these, **14** and **15**, were chosen for further preclinical development.

## Results and discussion

### Chemistry

**Scheme 1:**
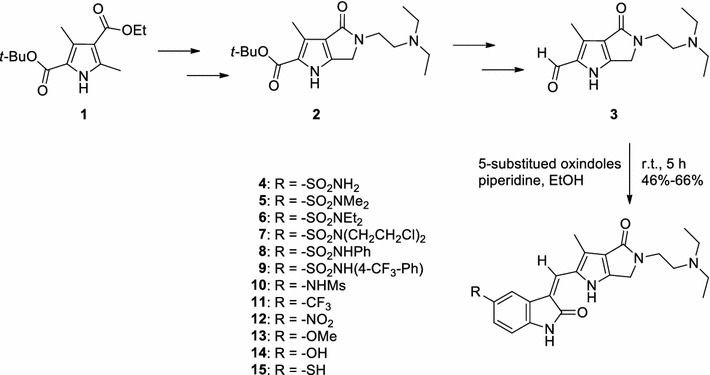
Synthesis of key intermediate **3** [19] and 5-substituted 2-pyrrolidone-fused (2-oxoindolin-3-ylidene)methylpyrrole derivatives

**Scheme 2:**
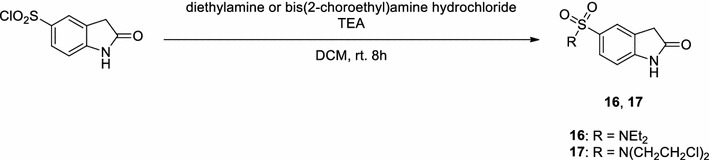
Synthesis of oxindoles **16** and **17**

Scheme 1 shows the approach used to synthesize the target products. Preparation of the key intermediate 5-(2-(diethylamino)ethyl)-3-methyl-4-oxo-1,4,5,6-tetrahydropyrrolo[3,4-*b*]pyrrole-2-carbaldehyde (**3**) was essentially performed as described in the literature [19]. Condensation of **3** with various 5-substitued oxindoles in the presence of piperidine at room temperature readily afforded target compounds **4**–**15** in the yield of 46–66%. Most of the requisite 5-substitued oxindoles were prepared by modifying methods described in the literature [26, 29–35] or were obtained commercially. The exceptions were *N*,*N*-diethyl-2-oxoindoline-5-sulfonamide (**16**) and *N*,*N*-bis(2-chloroethyl)-2-oxoindoline-5-sulfonamide (**17**), which were produced by direct amidation of 2-oxoindoline-5-sulfonyl chloride in dichloromethane at room temperature using triethylamine as a base [32] (Scheme 2). The resulting oxindoles **16** and **17** were used to synthesize the desired products **6** and **7**, respectively, as described in Scheme 1.

All the target compounds were isolated as free bases which were precipitated out during the synthesis. Compounds were purified by simply washing with EtOH. However, most cases required further purification by column chromatography (silica gel, 90:10:1 EtOAc–MeOH–TEA) with TEA to facilitate elution and to remove trace impurities with the exclusion of compound **7**. Purification of **7** by column chromatography using various solvent systems only led to rapid decomposition and then a string of unidentifiable spots from the eluent appeared in TLC. Our experiment results showed that analytically pure **7** could be obtainable smoothly by recrystallization from tetrahydrofuran (THF). All the structures of synthetic intermediates and products were determined by spectroscopy and specific data of high-resolution mass analysis (Additional file 1).

### Anti-proliferation activity

The in vitro anti-proliferation activity of synthetic compounds **4**–**15** and sunitinib (positive control) were evaluated in three different human cancer cell lines (human colon cancer cells HCT116, human non-small cell lung cancer cells NCI-H460, and human renal cell carcinoma 786-O) and a normal human fibroblast cell line Detroit 551. Table 1 summarizes the experimental results.

**Table 1:** Enzymatic and cellular inhibition activities of **4**–**15** and sunitinib


Compound	R	% inhibition of VEGFR-2 at 80 nM	IC_50_ (μM)/selective index (SI)			
			HCT116	NCI-H460	786-O	Detroit 551
Sunitinib	–	45	4.60 ± 0.23	7.51 ± 0.78	7.89 ± 0.60	9.48 ± 0.18
			1.32	0.81	0.76	
**4**	–SO_2_NH_2_	0	>10	>10	>10	>10
			nd	nd	nd	
**5**	–SO_2_NMe_2_	20	8.98 ± 0.92	>10	>10	>10
			>1.11	nd	nd	
**6**	–SO_2_NEt_2_	0	4.20 ± 0.57	>10	>10	>10
			>2.38	nd	nd	
**7**	–SO_2_N(CH_2_CH_2_Cl)_2_	46	3.65 ± 0.19	>10	>10	6.06 ± 0.40
			1.66	<0.61	<0.61	
**8**	–SO_2_NHPh	15	>10	>10	>10	>10
			nd	nd	nd	
**9**	–SO_2_NH(4-CF_3_-Ph)	0	>10	>10	>10	>10
			nd	nd	nd	
**10**	–NHMs	4	>10	>10	>10	>10
			nd	nd	nd	
**11**	–CF_3_	0	>10	7.22 ± 1.01	8.49 ± 0.46	>10
			nd	>1.39	>1.18	
**12**	–NO_2_	13	>10	6.61 ± 0.80	>10	>10
			nd	>1.51	nd	
**13**	–OMe	41	3.06 ± 0.67	6.37 ± 1.09	7.86 ± 0.30	>10
			>3.27	>1.57	>1.27	
**14**	–OH	58	2.83 ± 0.40	>10	>10	>10
			3.53	nd	nd	
**15**	–SH	57	2.34 ± 0.20	>10	>10	>10
			>4.27	nd	nd	

*nd* not detected

Compared to sunitinib, compounds **4**, **8**–**12** showed less activity against HCT116 cells (IC_50_ > 10 μM), indicating that electron-withdrawing groups (EWG) substituted at C(5) appeared detrimental to the anti-tumor activity of the 2-pyrrolidone-fused (2-oxoindolin-3-ylidene)methylpyrrole products [e.g., **11** (C(5)-CF_3_) and **12** (C(5)-NO_2_)]. However, introducing hydrogen bond donating (HBD) groups at C(5) in the 2-oxindole ring, e.g., **14** (C(5)-OH) and **15** (C(5)-SH), markedly inhibited HCT116 cells. From lowest to highest, the anti-proliferative activities against HCT116 cells based on the IC_50_ values were enhanced as follows: **15** (2.34 ± 0.20 μM) > **14** (2.83 ± 0.40 μM) > **13** (3.06 ± 0.67 μM) ≈ **7** (3.65 ± 0.19 μM) > **6** (4.20 ± 0.57 μM) ≈ sunitinib (4.60 ± 0.23 μM) > **5** (8.98 ± 0.92 μM). The presence and probably the appropriately positioning of HBD groups were apparently the main determinants of anti-proliferation potency. These experimental results indicated that C(5) substituted 2-pyrrolidone-fused (2-oxoindolin-3-ylidene)methylpyrroles against HCT116 cells had the descending order as follows: C(5)-HBD > C(5)-sulfonamide > C(5)-EWG. Regarding anti-proliferative effects on NCI-H460 cells, the IC_50_ values of **4**–**10**, **14**, and **15** were higher than 10 μM. Compounds **11**–**13** revealed approximately equal activity to sunitinib; however, their anti-proliferative activities did not significantly differ (p ≥ 0.05). For 786-O cells, the IC_50_ values of **4**–**10**, **12**, **14**, and **15** exceeded 10 μM. The order of anti-proliferative activities of **11**, **13** and sunitinib against 786-O cells was **13** ≈ sunitinib > **11**. Comparisons with our previously reported data confirmed the superior activity of **13** (C(5)-OMe) against 786-O cells to the corresponding C(5)-halogen 2-pyrrolidone-fused (2-oxoindolin-3-ylidene)methylpyrroles [19].

Since the proliferation of HCT116 cells is stimulated by HCT116-produced VEGF and VEGFR-1/2 via an autocrine mechanism, inhibiting VEGFR-1/2 of HCT116 cells with VEGFR-1/2 inhibitor AAL993 significantly decreases proliferation of HCT116 cells [36]. Table 1 shows that our experiments revealed a strong correlation between anti-proliferation activities of **4**–**15** against HCT116 cells and VEGFR-2 inhibition percentage at 80 nM.

Although NCI-H460 cells express both VEGF and VEGFR-2, proliferation of NCIH-460 cells is not promoted by VEGF/VEGFR-2 pathway [37]. Sunitinib has been approved for treating renal cell carcinoma (RCC); however, it inhibits RCC growth through an anti-angiogenesis mechanism rather than by directly targeting RCC cells [38]. Moreover, 786-O cells express VEGF and neuropilin-1 (NRP-1) rather than VEGFR-2. The VEGF promoted 786-O cell proliferation in an autocrine manner via VEGF/NRP-1 pathway [39]. Therefore, the IC_50_ values of most VEGFR-2 inhibiting compounds (**5**, **7**, **14**, **15**, and sunitinib) against either NCI-H460 or 786-O cells were higher than those of HCT116 cells. Interestingly, **11** (C(5)-CF_3_) showed cytotoxicity to both NCI-H460 and 786-O but not to HCT116 cells; **12** (C(5)-NO_2_) was toxic to NCI-H460; **13** (C(5)-OMe) was toxic to all three tested cancer cell lines. These experimental results suggest that the C(5) substituent replacement in this structural system significantly affected the selectivity of cancer cell growth inhibition.

Potential anticancer drug candidates should show greater selectivity for cancer cells compared with normal cells. Therefore, selectivity index (SI) values for synthetic products **4**–**15** as well as sunitinib were obtained in the three tested cancer lines (Table 1). For comparison, human normal fibroblast cells Detroit 551 were used as a control group. The SI values showed that all synthetic products except for **7** had high selectivity for tumor cells and, compared to sunitinib, even much lower toxicity to Detroit 551 cells. The toxic effects of C(5)-SO_2_N(CH_2_CH_2_Cl)_2_ substituent of **7** on Detroit 551 cells was evident and complex but nevertheless not yet completely understood. The likely explanation is that **7** contains a highly chemically reactive bis(2-chloroethyl)amino (–SO_2_N(CH_2_CH_2_Cl)_2_) similar to chlorambucil, which has clinic applications as a non-specific alkylating agent. Thus, its cytotoxic effect probably resulted from DNA damage via the formation of cross-links. In this study, **15** had particularly high selectivity to HCT116 cells (SI > 4.27 for **15** vs. 1.32 for sunitinib), and **13** had particularly high selectivity to NCI-H460 cells (SI > 1.57 for **13** vs. 0.81 for sunitinib) and 786-O cells (SI > 1.27 for **13** vs. 0.76 for sunitinib).

**Fig. 2:**
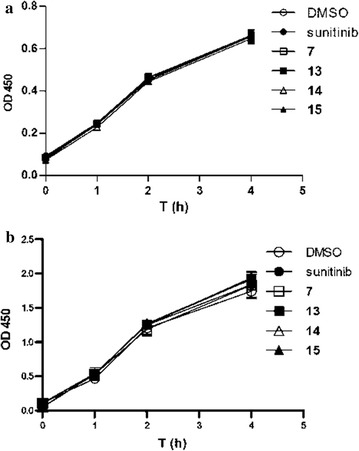
Acute cytotoxicity assay of **a** HCT116; **b** Detroit 551 incubated with DMSO (1%), sunitinib, **7**, and **13**–**15** (10 μM)

Since our newly synthesized products generally showed high selectivity against HCT116 cancer cell proliferation, the next experiment was performed to determine whether the inhibitory response resulted from acute cellular toxicity. Compounds **7** and **13**–**15** were then chosen to subject to acute cytotoxicity test on HCT116 cells through the WST-8 cell viability assay. Figure 2 shows the experimental results, which confirmed that neither our compounds nor sunitinib had acute cytotoxicity in the two tested cell lines.

**Fig. 3:**
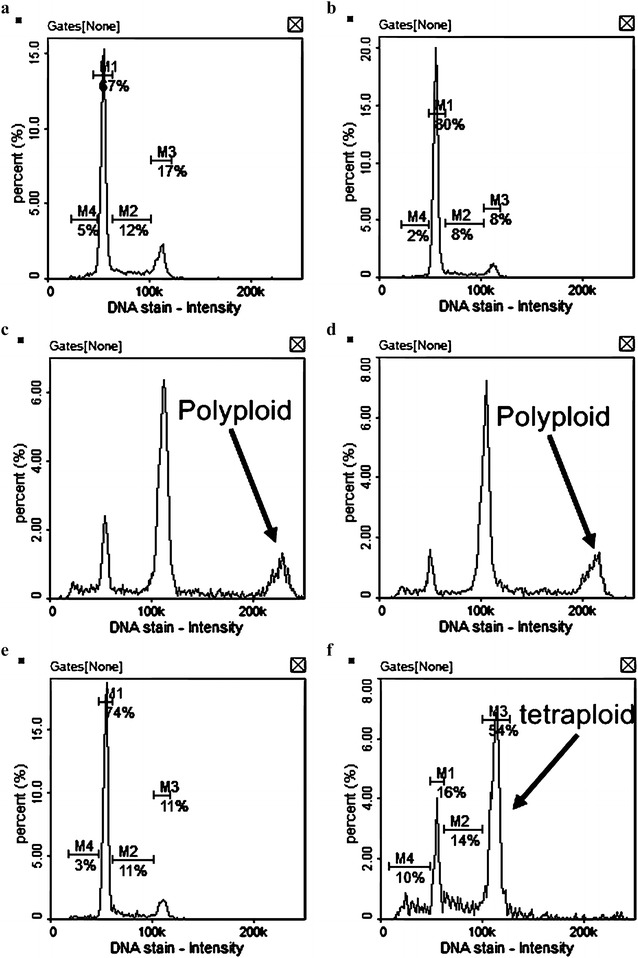
Cell cycle profiles of HCT-116 cells treated with **a** 1% DMSO (control); **b** sunitinib (5.0 μM); **c 7** (5.0 μM); **d 13** (3.0 μM); **e 14** (3.0 μM); **f 15** (3.0 μM) for 24 h. *M1* G0/G1phase, *M2* S phase, *M3* G2/M phase

Our previous works apparently showed that C(5)-halogen substituents of 2-pyrrolidone-fused (2-oxoindolin-3-ylidene)methylpyrroles affected the potency and cell cycle profiles of HCT116 cell [19]. For an improved understanding of these effects, this study performed further cell cycle analyses of **7**, **13**–**15**, and sunitinib (Fig. 3). The preliminary results showed that the cell cycle profiles of HCT116 cells incubated with **14** and sunitinib for 24 h caused G0/G1 cell cycle arrest. In contrast, the cell cycle profile of HCT116 cells incubated with **7** and **13** for 24 h displayed an increase in polyploid cells. Surprisingly, the cell cycle profile of HCT116 cells treated with **15** for 24 h showed an increase in tetraploid cells. Previous works had established that Inhibiting Aurora kinase obtained a polyploidal cell cycle profile [40–42]. Our previous studies proved that (2-oxoindolin-3-ylidene)methylpyrroles had great in vitro Aurora A kinase inhibition at 1.0 μM, and some of them revealed the inhibition of HCT116 cells proliferation via Aurora kinase inhibition. Our experiments again revealed a similar trend, i.e., 92.9% for **7**, 94.4% for **13**, and 93.6% for **15**, and 50.7% for sunitinib at 1.0 μM, respectively (Table 2). Therefore, we hypothesized that using compounds **7**, **13** and **15** to inhibit HCT116 cell proliferation might also inhibit Aurora kinase.

**Table 2:** In-vitro kinase inhibitory activities of **7**, **13**–**15**, and sunitinib


Compound	R	IC_50_ (nM)		% inhibition of Aurora A at 1.0 μM
		VEGFR-2	PDGFRβ	
Sunitinib	–	151.8	94.5	50.7
**7**	–SO_2_N(CH_2_CH_2_Cl)_2_	23.7	63.2	94.4
**13**	–OMe	47.8	76.2	92.9
**14**	–OH	25.9	28.5	93.6
**15**	–SH	14.6	12.1	96.4

In summary, the experiments in this study suggested that substituents at C(5) markedly influenced the anti-proliferation activity and selectivity of synthetic derivatives of 2-pyrrolidone-fused (2-oxoindolin-3-ylidene)methylpyrrole. Additionally, hydrogen bond donor substituents at C(5) significantly affected the potency and selectivity of anti-proliferation activity.

### Kinase inhibitory assay**s**

Next, the VEGFR-2 phosphorylation inhibitory activities of the newly synthesized compounds were evaluated. The experimental results in Table 1 show that the VEGFR-2 inhibitory activities of compounds **4**, **8**, **9**, and **11** at concentrations of 80 nM did not differ from that of the 1% DMSO (control). However, compounds **5**, **6**, and **12** at the same concentration revealed 13–20% inhibition; **7** and **13** demonstrated approximately equal inhibition percentage to sunitinib; and **14** and **15** exhibited the most potent inhibitory activity. Therefore, IC_50_ values of compounds **7** and **13**–**15** were further evaluated to assess their activities against VEGFR-2, PDGFRβ, and Aurora A kinase.

Sun et al. showed that C(5)-SO_2_NH_2_ at (2-oxoindolin-3-ylidene)methylpyrroles improved VEGFR-2 inhibition [21]. A pharmacophore model of oxindole analog binding at the FGFR1 binding site generated from virtual screen results in a study by Kammasud then revealed that introduction of a phenyl hydrazide motif to C(5) of oxindoles proved to be the best possible to allow additional hydrogen bonding interactions with ATP site of receptor tyrosine kinases (RTKs), such as FGFR-1, VEGFR-2, PDGFRβ, and EGFR [20]. In our investigation, compounds **4** (C(5)-SO_2_NH_2_), **5** (C(5)-SO_2_NMe_2_), **6** (C(5)-SO_2_NEt_2_), **8** (C(5)-SO_2_NHPh), and **9** (C(5)-SO_2_NH(4-CF_3_-Ph)) showed disappointing activities or only mediocre improvement in VEGFR-2 inhibition; however, **7** (C(5)-SO_2_N(CH_2_CH_2_Cl)_2_) displayed evident improvement. These experimental results suggest that ligand–protein binding affinity between VEGFR-2 and 2-pyrrolidone-fused (2-oxoindolin-3-ylidene)methylpyrroles is probably not be enhanced by either C(5)-SO_2_NH_2_, C(5)-SO_2_NHPh or C(5)-SO_2_N(alkyl)_2_, with the exception of **7** (C(5)-SO_2_N(CH_2_CH_2_Cl)_2_), the discrepancy of which already discussed.

Since our previously reported C(5)-halogen substituted 2-pyrrolidone-fused (2-oxoindolin-3-ylidene)methylpyrrole derivatives showed fairly potent inhibiting effects on VEGFR-2 (35–64% inhibition at 50 nM) [19], our next objective was bioisosteric replacement of the C(5)-halogens with an electron-withdrawing C(5)-CF_3_. Unfortunately, **11** (C(5)-CF_3_) had no inhibitory activity against VEGFR-2 at 80 nM.

The effect of a C(5)-OMe substituent of indoline-2-one scaffold on kinase inhibitory activity and selectivity is highly dependent on the C(3) substituents of indoline-2-one [22, 26]. Interestingly, our study showed that compound **13** (C(5)-OMe) substantially improved VEGFR-2 inhibition but not so noticeable in PDGFRβ inhibition. A more or less similar effect could be observed in **7** (C(5)-SO_2_N(CH_2_CH_2_Cl)_2_).

As Table 2 shows, in comparison to sunitinib, compounds **7** and **13** had six- and threefold lower IC_50_ values for VEGFR-2, respectively. Moreover, compared to their C(5)-OMe analog **13**, compounds **14** (C(5)-OH) and **15** (C(5)-SH) even showed a two- and a fourfold decrease in IC_50_ values, respectively. On the other hand, the inhibiting activities of **7** and **13** in PDGFRβ were slightly more potent than those of sunitinib; however, **14** and **15** had a three- and an eightfold decrease in IC_50_ values for inhibiting PDGFRβ, respectively. Thus, both C(5)-OH and C(5)-SH substituents could significantly improve the activity of 2-pyrrolidone-fused (2-oxoindolin-3-ylidene)methylpyrroles in the inhibition of both VEGFR-2 and PDGFRβ. In accordance with Kammasud, we hypothesized that groups C(5)-OH and C(5)-SH probably produced favorable potency of **14** and **15** by providing additional hydrogen bonding interactions with ATP site of RTKs.

The results once again revealed a similar trend, i.e., different C(5) substitutions markedly affect the biochemical activities against VEGFR-2 and PDGFRβ. In summary, hydrogen-bond-donating (HBD) substituent at C(5) could greatly enhance inhibitory potency against both VEGFR-2 and PDGFRβ. These experimental results suggest that the influence of C(3) substituent to the C(5)-HBD substituted indoline-2-one scaffold needs further study.

### In-vitro tube formation assay

**Fig. 4:**
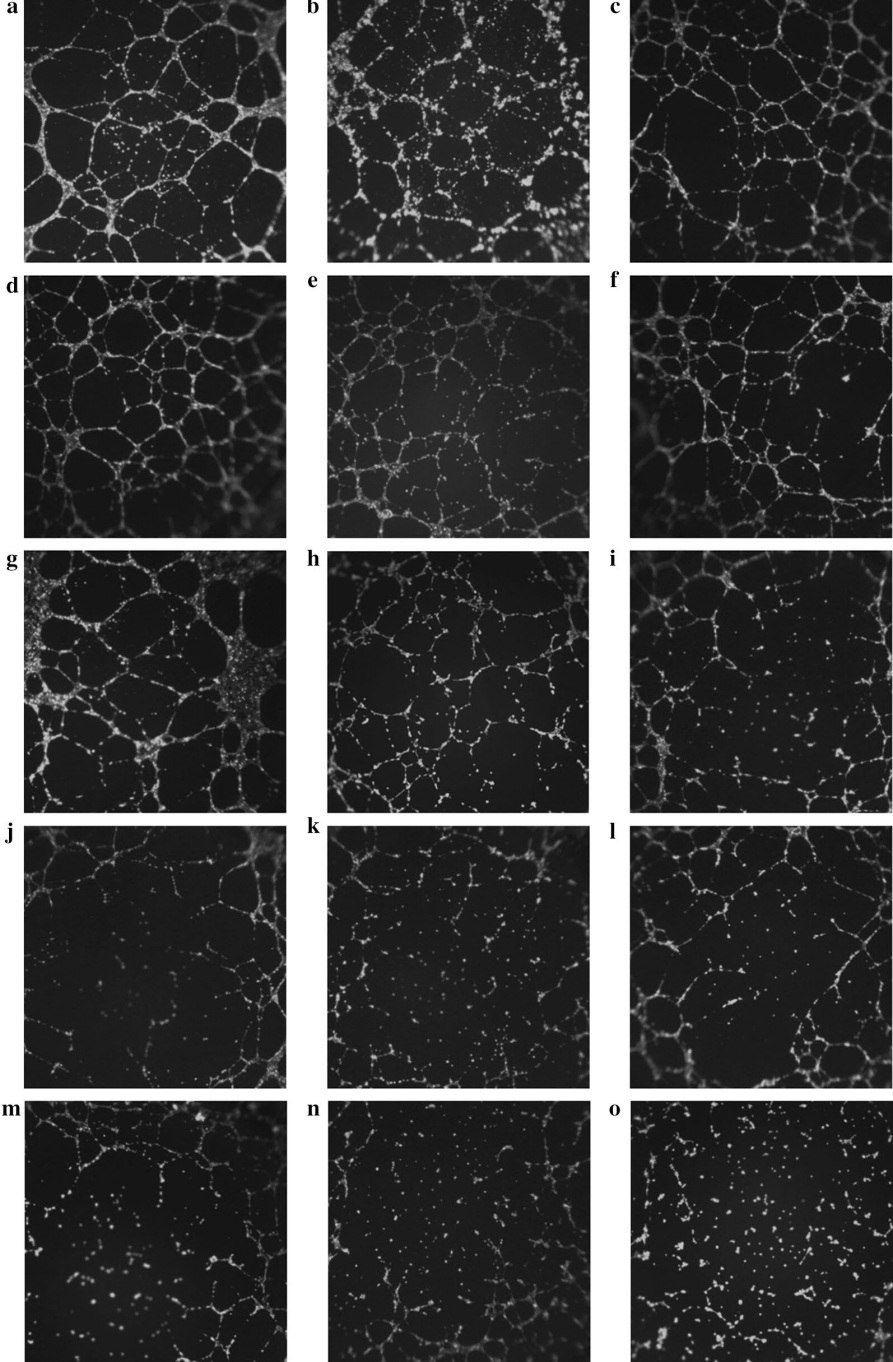
Compounds **7**, **13**–**15**, and sunitinib inhibited tube formation induced by VEGF. **a** Solvent control; **b** VEGF (10 ng/ml) and 0.1 μM sunitinib; **c** VEGF (10 ng/ml) and 0.10 μM **7**; **d** VEGF (10 ng/ml) and 0.10 μM **13**; **e** VEGF (10 ng/ml) and 0.10 μM **14**; **f** VEGF (10 ng/ml) and 0.10 μM **15**; **g** VEGF (10 ng/ml) and 0.50 μM sunitinib; **h** VEGF (10 ng/ml) and 0.50 μM **7**; **i** VEGF (10 ng/ml) and 0.50 μM **13**; **j** VEGF (10 ng/ml) and 0.50 μM **14**; **k** VEGF (10 ng/ml) and 0.50 μM **15**; **l** VEGF (10 ng/ml) and 1.0 μM sunitinib; **m** VEGF (10 ng/ml) and 1.0 μM **7**; **n** VEGF (10 ng/ml) and 1.0 μM **13**; **o** VEGF (10 ng/ml) and 1.0 μM **14**

**Fig. 5:**
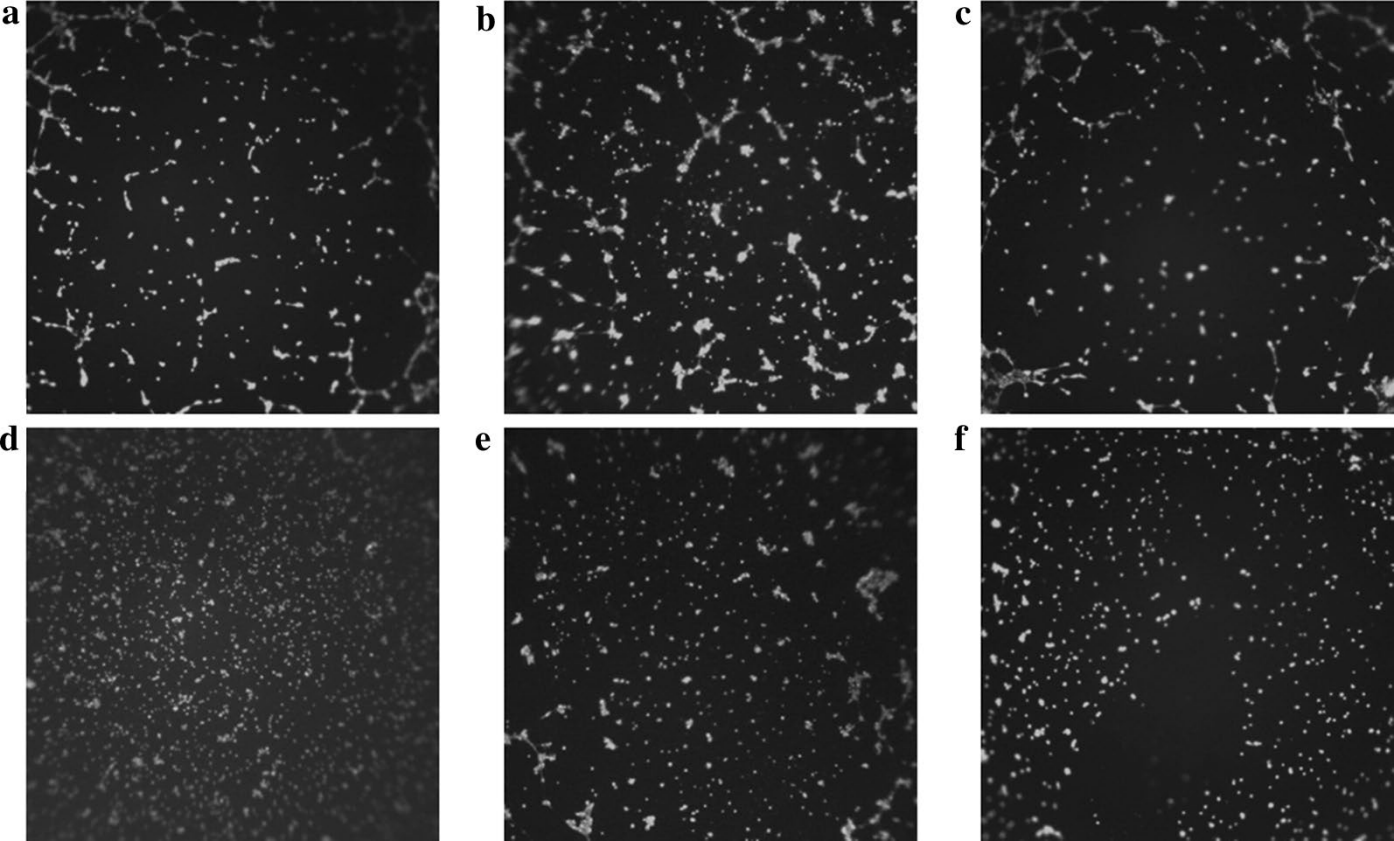
Compounds **7**, **13**–**15**, and sunitinib inhibited tube formation induced by VEGF. **a** VEGF (10 ng/ml) and 1.0 μM **15**; **b** VEGF (10 ng/ml) and 2.0 μM sunitinib; **c** VEGF (10 ng/ml) and 2.0 μM **7**; **d** VEGF (10 ng/ml) and 2.0 μM **13**; **e** VEGF (10 ng/ml) and 2.0 μM **14**; **f** VEGF (10 ng/ml) and 2.0 μM **15**

In-vitro VEGF-induced tube formation inhibitory activity of **7**, **13**–**15**, and sunitinib were tested by Matrigel tube formation assay using ibidi μ-Slide angiogenesis kit. Figures 4 and 5 shows the photographs of Matrigel tube formation assays of control and tested compounds at 2.0, 1.0, 0.50 and 0.10 μM. Under these conditions, the density of tube-like structures was substantially reduced. Compounds **7** and **13**–**15** showed distinctly higher tube formation inhibitory activity than the reference drug sunitinib. Compared to sunitinib, **7** and **13** had twofold higher potency in inhibiting in vitro tube formation than sunitinib in terms of IC_50_ (Table 3). The more potent **14** and **15** were with IC_50_ roughly 2.5- and threefold stronger than sunitinib, respectively. These experimental results agree well with those for VEGFR-2 kinase inhibitory assays, suggesting that our synthetic compounds inhibited the in vitro tube formation via VEGFR-2 inhibition.

**Table 3:** Inhibition activities of **7**, **13**–**15**, and sunitinib against in vitro tube formation


Compound	R	IC_50_ (μM)
		Area
Sunitinib	–	1.54 ± 0.08
**7**	–SO_2_N(CH_2_CH_2_Cl)_2_	0.76 ± 0.11
**13**	–OMe	0.74 ± 0.16
**14**	–OH	0.62 ± 0.07
**15**	–SH	0.53 ± 0.11

### Molecular modeling

**Fig. 6:**
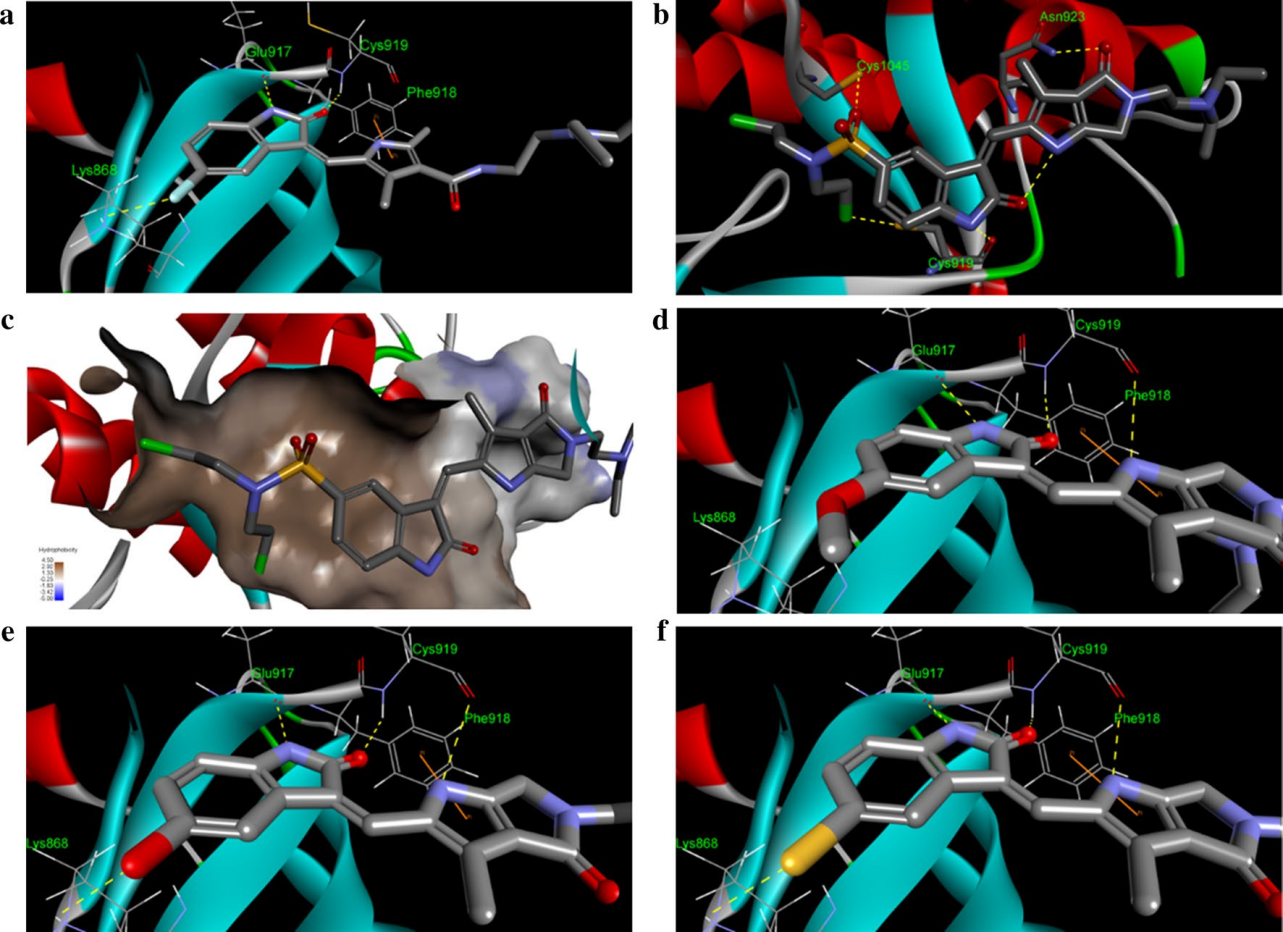
Sunitinib, **7**, and **13**–**15** are docked into the active site of VEGFR-2 (PDB ID: 4AGD) in 3-dimentional structure. **a** Sunitinib in VEGFR-2; **b 7** in VEGFR-2; **c 7** in VEGFR-2 (the active site of VEGFR-2 was shown in hydrophobicity maps); **d 13** in VEGFR-2; **e 14** in VEGFR-2, **f 15** in VEGFR-2. Compounds are showed in *sticks*; hydrogen bonds are shown as *dashed yellow line*; pi–pi interaction is shown in *orange line*; *shades of brown* indicate regions of high hydrophobicity; *shades of white* indicate regions of neutral, *shades of blue* indicate regions of low hydrophobicity

The kinase inhibitory assays revealed that the VEGFR-2 and in vitro tube formation inhibitory activities of the synthetic compounds **7** and **13**–**15** exceeded that of sunitinib, and the C(5) substituents of 2-pyrrolidone-fused (2-oxoindolin-3-ylidene)methylpyrrole were critical for VEGFR-2 inhibitory activity. For further clarification of these results, sunitinib, **7**, and **13**–**15** were examined and compared by docking into the ATP-binding site of VEGFR-2 (PDB ID: 4AGD) using Discovery Studio LibDock [43]. LibDock is a method placing the generated ligand conformations into the protein active site based on polar and apolar interaction sites (hotspot). Figure 6 shows the predicted binding modes of sunitinib, **7**, and **13**–**15**. Interestingly, the modeling results for compound **7** differed from those of compounds **13**–**15** in that **7** formed four hydrogen bonds with VEGFR-2: the Cl of C(5)-SO_2_N(CH_2_CH_2_Cl)_2_ and the NH of the oxindole scaffold of **7** formed hydrogen bonds with the same Cys919, the oxygen atom of C(5)-SO_2_N(CH_2_CH_2_Cl)_2_ with Cys1045, and the oxygen atom of pyrrolidone (C(4′)) with Asn923 (Fig. 6b). The docking results further showed that C(5)-SO_2_N(CH_2_CH_2_Cl)_2_ of **7** was laid in the hydrophobic pocket of the VEGFR-2 active site (Fig. 6c). The above experimental results might explain why compound **7** had the most potent VEGFR-2 inhibiting effects among **4**–**9**. In Fig. 6d–f, the predicted binding modes of highly active compounds **13**–**15** reveal that each of them formed three hydrogen bonds with Lys868, Glu917, and Cys919, respectively. Additionally, compounds **13**–**15** all formed pi–pi interactions between their pyrrole-scaffolds and Phe918 of VEGFR-2 (Fig. 6d–f). Most notably, C(5)-OH of **14** and C(5)-SH of **15** formed hydrogen bonds with Lys686 of VEGFR-2 (Fig. 6e, f) while C(5)-OMe of **13**, the methyl ether of **14** but with much lower activity, did not show any interaction with Lys868 due to the blockade of –OMe to hydrogen bond formation. These experimental results indicate that C(5)-HBD of 2-pyrrolidone-fused (2-oxoindolin-3-ylidene)methylpyrrole derivatives have important inhibiting effects on VEGFR-2 activity, and compounds **14** and **15** proved to be the case.

## Conclusions

The novel series of 2-pyrrolidone-fused (2-oxoindolin-3-ylidene)methylpyrrole derivatives with various C(5) substitutions synthesized in our laboratory showed notable cellular and enzymatic anti-tumor activities. Several of these derivatives had superior inhibitory activity against VEGFR-2 and PDGFRβ compared to sunitinib. Among them, **14** (C(5)-OH) and **15** (C(5)-SH) possessed the highest potency and the highest selectivity in HCT116 cells. The preliminary results in further pharmacokinetic studies of compounds **14** and **15** were satisfactory. Detailed pharmacological and pharmacokinetic studies are in progress and will be reported in future works.

## Experiment section

### Chemistry

All the chemicals were purchased from Aldrich-Sigma Chemical Company (St.  Louis, MO, USA) and Alfa-Aesar Chemical Company (Lancashire, Heysham,  England) and used without further purification. All reactions were routinely monitored by TLC on Merck F_254_ silica gel plates. Silica gel (70–230 mesh, Silicacycle) was used for column chromatography. The ^1^H- and ^13^C-NMR spectra were determined on an Agilent Varian-400 NMR (Agilent Technologies, Santa Clara, CA, USA) instrument in CDCl_3_, acetone-*d*_6_, methanol-*d*_4_, or acetic acid-*d*_6_ unless otherwise noted. Chemical shifts (*δ*) were expressed as parts per million (ppm) downfield from tetramethylsilane (TMS) as the internal standard (*σ* 0.00), and coupling constants (*J*) were given in hertz (Hz). High-resolution mass spectra (HRMS) using a Bruker Impact HD (ESI) were performed in the Instrument Center of the Ministry of Science and Technology at the National Chiao-Tung University, Taiwan. Dry tetrahydrofuran (THF) was freshly distilled from lithium aluminum hydride (LAH) before use. All the other solvents were obtained from commercial sources and purified before use if necessary. Images were acquired with a Leica DM1000 LED microscope (Leica Microsystems, Wetzlar, Hessen, Germany). UV–VIS spectra were recorded on a Thermo Multiskan Go Microplate spectrophotometer (Thermo Fisher Scientific, Waltham, MA, USA). IR spectra were registered on a Thermo Nicolet iS5 FT-IR spectrometer (Thermo Fisher Scientific, Waltham, MA, USA) with attenuated total reflection (ATR) method. The purities of the final compounds were all greater than 95% as determined by analytical reverse-phase HPLC.

### General procedure for the preparation of compounds **4**–**15**

The key intermediate **3** for preparation of target compounds was synthesized according to the method reported previously [19]. To each stirred solution containing **3** (263 mg, 1.00 mmol) in EtOH (15 ml) was added dropwise a solution of 5-substituted oxindole (1.00 mmol) in EtOH (2 ml) and then piperidine (0.1 ml) was added. After stirring at room temperature for 6 h, the precipitate formed was filtrated, washed with EtOH, and purified by column chromatography (silica gel, 90:10:1 EtOAc–MeOH–TEA).

#### (*Z*)-3-((5-(2-(diethylamino)ethyl)-3-methyl-4-oxo-1,4,5,6-tetrahydropyrrolo[3,4-*b*]pyrrol-2-yl)methylene)-2-oxoindoline-5-sulfonamide (**4**)

The requisite oxindole-5-sulfonamide, for condensation with **3** to form target compound **4**, was prepared in 60% yield by a modified method of amidation of 2-oxoindoline-5-sulfonyl chloride with ammonium hydroxide solution [32].

Yield of **4**: 58%, orange solids. Mp: 252–255 °C, UV λ_max_ (MeOH), nm (logɛ): 418 (4.21). IR (ATR), cm^−1^: 3239, 2976, 1600, 1562. ^1^H-NMR (400 MHz, acetic acid-*d*_4_) *δ*, ppm: 8.26 (d, 1H, *J* = 1.2 Hz Ar**H**), 7.74–7.77 (m, 2H, –C=C**H**–, Ar**H**), 7.12 (d, 1H, *J* = 8.4 Hz, Ar**H**), 4.56 (s, 2H, Ar–C**H**_**2**_), 4.00 (t, 2H, *J* = 5.6 Hz, –NC**H**_**2**_CH_2_–), 3.50 (t, 2H, *J* = 5.6 Hz, –NCH_2_C**H**_**2**_–), 3.37 (q, 4H, *J* = 7.2 Hz, –N(C**H**_**2**_CH_3_)_2_), 2.51 (s, 3H, Ar–C**H**_**3**_), 1.31 (t, 6H, *J* = 7.2 Hz, –N(CH_2_C**H**_**3**_)_2_). ^13^C-NMR (100 MHz, acetic acid-*d*_4_) *δ*, ppm: 171.5, 169.5, 150.2, 142.0, 137.7, 133.8, 128.1, 127.4, 126.9, 126.5, 121.3, 117.9, 116.5, 111.2, 51.8, 48.1, 45.7, 39.5, 9.9, 8.7. HRMS *m/z* (ESI): calcd., 458.1869.1744 [M + H]^+^; found, 458.1857 [M + H]^+^.

#### (*Z*)-3-((5-(2-(diethylamino)ethyl)-3-methyl-4-oxo-1,4,5,6-tetrahydropyrrolo[3,4-*b*]pyrrol-2-yl)methylene)-*N*,*N*-dimethyl-2-oxoindoline-5-sulfonamide (**5**)

The requisite *N*,*N*-dimethyl-2-oxoindoline-5-sulfonamide, for condensation with **3** to form target compound **5**, was prepared in 74% yield by a modified method of amidation of 2-oxoindoline-5-sulfonyl chloride with dimethylamine in methanol [32].

Yield of **5**: 56%, orange solids. Mp: 244–247 °C, UV λ_max_ (MeOH), nm (logɛ): 428 (4.53). IR (ATR), cm^−1^: 2971, 1657, 1586. ^1^H-NMR (400 MHz, acetic acid-*d*_4_) *δ*, ppm: 8.19 (d, 1H, *J* = 1.6 Hz, Ar**H**), 7.86 (s, 1H, –C=C**H**–), 7.63 (dd, 1H, *J* = 8.0, 1.6 Hz, Ar**H**), 7.18 (d, 1H, *J* = 1.6 Hz, Ar**H**), 4.57 (s, 2H, Ar–C**H**_**2**_), 3.96 (t, 2H, *J* = 6.0 Hz, –NC**H**_**2**_CH_2_–), 3.50 (t, 2H, *J* = 6.0 Hz, –NCH_2_C**H**_**2**_–), 3.39 (q, 4H, *J* = 7.2 Hz, –N(C**H**_**2**_CH_3_)_2_), 2.68 (s, 6H, –N(C**H**_**3**_)_2_), 2.50 (s, 3H, Ar–C**H**_**3**_), 1.33 (t, 6H, *J* = 7.2 Hz, –N(CH_2_C**H**_**3**_)_2_). ^13^C-NMR (100 MHz, acetic acid-*d*_4_) *δ*, ppm: 171.5, 169.5, 150.2, 142.4, 133.8, 129.7, 128.2, 128.1, 127.7, 127.1, 121.3, 119.5, 116.4, 111.3, 51.7, 48.5, 48.0, 39.4, 38.3, 10.0, 8.7. HRMS *m/z* (ESI): calcd., 486.2172 [M + H]^+^; found, 486.2170 [M + H]^+^.

#### (*Z*)-3-((5-(2-(diethylamino)ethyl)-3-methyl-4-oxo-1,4,5,6-tetrahydropyrrolo[3,4-*b*]pyrrol-2-yl)methylene)-*N*,*N*-diethyl-2-oxoindoline-5-sulfonamide (**6**)

2-Oxoindoline-5-sulfonyl chloride (2.32 g, 10.0 mmol), prepared by a modified method [32], was suspended in dichloromethane (20 ml). The resulting mixture was added dropwise a solution of triethylamine (1.21 g, 12.0 mmol) in dichloromethane (5 ml) and then a solution of diethylamine (0.90 g, 12.0 mmol) in dichloromethane (5 ml) was added. After stirring at room temperature for 8 h, the precipitate formed was filtrated, washed with dichloromethane, and purified by column chromatography (silica gel, 1:4 EtOAc–Hexane) to yield 1.90 g (71%) of *N*,*N*-diethyl-2-oxoindoline5-sulfonamide (**16**) as pale yellow crystals. Mp: 155–156 °C, UV λ_max_ (MeOH), nm (logɛ): 289 (4.64). IR (ATR), cm^−1^: 3159, 1707, 1615. ^1^H NMR (400 MHz, methanol-*d*_4_) *δ*, ppm: 7.70 (d, 1H, *J* = 6.8 Hz, Ar**H**), 7.69 (s, 1H, Ar**H**), 7.02 (d, 1H, *J* = 6.8 Hz, Ar**H**) 3.62 (s, 2H, ArC**H**_**2**_), 3.21 (q, 4H, *J* = 7.2 Hz, –N(C**H**_**2**_CH_3_)_2_), 1.13 (t, 6H, *J* = 7.2 Hz, –N(CH_2_C**H**_**3**_)_2_). ^13^C-NMR (100 MHz, methanol-*d*_4_) *δ*, ppm: 179.6, 148.9, 134.7, 129.1, 128.2, 124.6, 110.6, 47.8, 43.4, 14.7. HRMS *m/z* (EI): calcd., 268.0882 [M]^+^; found, 268.0872 [M]^+^.

*N*,*N*-Diethyl-2-oxoindoline5-sulfonamide (**16**) obtained from above was used to condense with **3** to afford target compound **6** as described in general synthesis. Yield of **6**: 57%, orange solids. Mp: 245–247 °C, UV λ_max_ (MeOH), nm (logɛ): 429 (4.54). IR (ATR), cm^−1^: 2968, 1659, 1589. ^1^H-NMR (400 MHz, acetic acid-*d*_4_) *δ*, ppm: 8.20 (d, 1H, *J* = 2.0 Hz, Ar**H**), 7.79 (s, 1H, –C=C**H**–), 7.63 (dd, 1H, *J* = 8.0, 2.0 Hz, Ar**H**), 7.11 (d, 1H, *J* = 8.0 Hz, Ar**H**), 4.52 (s, 2H, Ar–C**H**_**2**_), 3.92 (t, 2H, *J* = 7.2 Hz, –NC**H**_**2**_CH_2_–), 3.47 (t, 2H, *J* = 7.2 Hz, –NCH_2_C**H**_**2**_–), 3.36 (q, 4H, *J* = 7.2 Hz, –N(C**H**_**2**_CH_3_)_2_), 3.21 (q, 4H, *J* = 7.2 Hz, –N(C**H**_**2**_CH_3_)_2_), 2.47 (s, 3H, Ar–C**H**_**3**_), 1.31 (t, 6H, *J* = 7.2 Hz, –N(CH_2_C**H**_**3**_)_2_), 1.10 (t, 6H, *J* = 7.2 Hz, –N(CH_2_C**H**_**3**_)_2_). ^13^C-NMR (100 MHz, acetic acid-*d*_4_) *δ*, ppm: 171.4, 169.4, 150.1, 142.1, 134.7, 133.8, 128.1, 127.5, 127.2, 127.0, 121.3, 118.8, 116.5, 111.4, 51.7, 48.5, 48.1, 43.2, 39.4, 14.7, 10.0, 8.7. HRMS *m/z* (ESI): calcd., 514.2497 [M + H]^+^; found, 514.2483 [M + H]^+^.

#### (*Z*)-*N*,*N*-Bis(2-chloroethyl)-3-((5-(2-(diethylamino)ethyl)-3-methyl-4-oxo-1,4,5,6-tetrahydropyrrolo[3,4-*b*]pyrrol-2-yl)methylene)-2-oxoindoline-5-sulfonamide (**7**)

2-Oxoindoline-5-sulfonyl chloride (2.32 g, 10.0 mmol), prepared by a modified method [32], and bis(2-choroethyl)amine hydrochloride (2.14 g, 12.0 mmol) were suspended in dichloromethane (20 ml) and then was added dropwise a solution of triethylamine (2.23 g, 22.0 mmol) in dichloromethane (10 ml). After stirring at room temperature for 8 h, the precipitate formed was filtrated, washed with dichloromethane, and purified by column chromatography (silica gel, 1:4 EtOAc–Hexane). 2.13 g (63%) of *N*,*N*-bis(2-chloroethyl)-2-oxoindoline-5-sulfonamide **(17)** as pale yellow solids. Mp: 193–195 °C, UV λ_max_ (MeOH), nm (logɛ): 289 (4.60). IR (ATR), cm^−1^: 3157, 1704, 1615. ^1^H NMR (400 MHz, CDCl_3_) δ, ppm: 7.76 (d, 1H, *J* = 8.4, Ar**H**), 7.71 (s, 1H, Ar**H**), 6.98 (d, 1H, *J* = 8.4, Ar**H**), 3.70 (t, 4H, *J* = 7.0 Hz, –N(CH_2_ **CH**_**2**_Cl)_2_), 3.62 (s, 2H, ArC**H**_**2**_) 3.49 (t, 4H, *J* = 7.0 Hz, –N(C**H**_**2**_CH_2_Cl)_2_). ^13^C-NMR (100 MHz, dmso-*d*_6_) *δ*, ppm: 176.4, 148.2, 130.3, 128.0, 123.3, 117.5, 113.5, 109.2, 50.3, 42.3, 39.5.

*N*,*N*-Bis(2-chloroethyl)-2-oxoindoline-5-sulfonamide **(17)** obtained from above was used to condense with **3** to afford target compound **7** as described in general synthesis. Purification of **7** was performed by recrystallization from THF. Yield of **7**: 54%, orange solids. Mp: 236–237 °C, UV λ_max_ (MeOH), nm (logɛ): 428 (4.58). IR (ATR), cm^−1^: 2917, 1651, 1574. ^1^H-NMR (400 MHz, acetic acid-*d*_4_) *δ*, ppm: 8.29 (s, 1H, –C=C**H**–), 7.87 (s, 1H, Ar**H**), 7.73 (d, 1H, *J* = 8.4 Hz, Ar**H**,), 7.18 (d, 1H, *J* = 8.4, Ar**H**), 4.60 (s, 2H, Ar–C**H**_**2**_), 3.98 (2H, *J* = 7.2 Hz, –NC**H**_**2**_CH_2_–) 3.73 (t, 4H, *J* = 7.2 Hz, –N(CH_2_C**H**_**2**_Cl)_2_), 3.53 (t, 6H, *J* = 7.2 Hz, –NCH_2_C**H**_**2**_–, –N(C**H**_**2**_CH_2_Cl)_2_), 3.39 (q, 4H, *J* = 7.2 Hz, –N(C**H**_**2**_CH_3_)_2_), 2.50 (s, 3H, Ar–C**H**_**3**_), 1.31 (t, 6H, *J* = 7.2 Hz, –N(CH_2_C**H**_**3**_)_2_). ^13^C-NMR (100 MHz, acetic acid-*d*_4_) *δ*, ppm: 171.5, 169.5, 150.4, 142.6, 133.8, 133.4, 128.4, 127.9, 127.6, 127.3, 121.4, 119.0, 116.3, 111.5, 52.2, 51.8, 48.5, 48.1, 43.1, 39.4, 10.0, 8.7. HRMS *m/z* (ESI): calcd., 582.1718 [M + H]^+^; found, 582.1703 [M + H]^+^.

#### (*Z*)-3-((5-(2-(Diethylamino)ethyl)-3-methyl-4-oxo-1,4,5,6-tetrahydropyrrolo[3,4-*b*]pyrrol-2-yl)methylene)-2-oxo-*N*-phenylindoline-5-sulfonamide (**8**)

The requisite 2-oxo-*N*-phenylindoline-5-sulfonamide, for condensation with **3** to form target compound **8**, was prepared in 83% yield by a modified method of amidation of 2-oxoindoline-5-sulfonyl chloride with aniline [32].

Yield of **8**: 46%, orange solids. Mp: 225–228 °C, UV λ_max_ (MeOH), nm (logɛ): 429 (4.47). IR (ATR), cm^−1^: 3213, 2963, 1667, 1557. ^1^H-NMR (400 MHz, acetic acid-*d*_4_) *δ*, ppm: 8.12 (d, 1H, *J* = 1.6, Ar**H**), 7.71 (s, 1H, –C=C**H**–), 7.59 (dd, 1H, *J* = 8.4, 1.6 Hz, Ar**H**), 7.22–7.14, (m, 5H, Ar**H**), 7.02 (d, 1H, *J* = 8.4 Hz, Ar**H**), 4.56 (s, 2H, Ar–C**H**_**2**_), 3.95 (t, 2H, *J* = 6.0 Hz, –NC**H**_**2**_CH_2_–), 3.48 (t, 2H, *J* = 6.0 Hz, –NCH_2_C**H**_**2**_–), 3.37 (q, 4H, *J* = 7.0 Hz, –N(C**H**_**2**_CH_3_)_2_), 2.48 (s, 3H, Ar–C**H**_**3**_), 1.31 (t, 6H, *J* = 7.0 Hz, –N(CH_2_C**H**_**3**_)_2_). ^13^C-NMR (100 MHz, acetic acid-*d*_4_) *δ*, ppm: 171.3, 169.4, 150.2, 142.3, 138.4, 133.9, 133.7, 130.2, 128.1, 127.5, 127.3, 126.8, 125.9, 122.2, 121.3, 118.8, 116.2, 111.1, 51.7, 48.5, 48.1, 39.4, 10.0, 8.7. HRMS *m/z* (ESI): calcd., 534.2182 [M + H]^+^; found, 534.2181 [M + H]^+^.

#### (*Z*)-3-((5-(2-(Diethylamino)ethyl)-3-methyl-4-oxo-1,4,5,6-tetrahydropyrrolo[3,4-*b*]pyrrol-2-yl)methylene)-2-oxo-*N*-(4-(trifluoromethyl)phenyl)indoline-5-sulfonamide (**9**)

The requisite 2-oxo-*N*-(4-(trifluoromethyl)phenyl)indoline-5-sulfonamide, for condensation with **3** to form target compound **9**, was prepared in 77% yield by a modified method of amidation of 2-oxoindoline-5-sulfonyl chloride with 4-(trifluoromethyl)aniline [32].

Yield of **9**: 46%, orange solids. Mp: 231–234 °C, UV λ_max_ (MeOH), nm (logɛ): 429 (4.32). IR (ATR), cm^−1^: 3162, 1634, 1582. ^1^H-NMR (400 MHz, acetic acid-*d*_4_) *δ*, ppm: ^1^H-NMR (400 MHz, acetic acid-*d*_4_) *δ*, ppm: 8.21 (s, 1H, Ar**H**), 7.75 (s, 1H, –C=C**H**–), 7.68 (dd, 1H, *J* = 8.0, 1.6 Hz, Ar**H**), 7.52 (d, 2H, *J* = 8.4 Hz, Ar**H**), 7.34 (d, 2H, *J* = 8.4 Hz, Ar**H**), 7.05 (d, 1H, *J* = 8.0 Hz, Ar**H**), 4.58 (s, 2H, Ar-C**H**_**2**_), 3.97 (t, 2H, *J* = 7.2 Hz, –NC**H**_**2**_CH_2_–), 3.50 (t, 2H, *J* = 7.2 Hz, –NCH_2_C**H**_**2**_–), 3.38 (q, 4H, *J* = 7.2 Hz, –N(C**H**_**2**_CH_3_)_2_), 2.50 (s, 3H, Ar–C**H**_**3**_), 1.32 (t, 6H, *J* = 7.2 Hz, –N(CH_2_C**H**_**3**_)_2_).

^13^C-NMR (100 MHz, acetic acid-*d*_4_) *δ*, ppm: 171.4, 169.5, 150.4, 142.7, 142.3, 133.7, 128.3, 127.6, 127.5, 127.4, 127.4, 127.1, 126.9, 126.6, 126.6, 124.0, 121.4, 120.6, 118.8, 111.3, 51.8, 48.2, 48.1, 39.4, 39.5, 10.0, 8.7. HRMS *m/z* (ESI): calcd., 602.2060 [M + H]^+^; found, 602.2043 [M + H]^+^.

#### (*Z*)-*N*-(3-((5-(2-(Diethylamino)ethyl)-3-methyl-4-oxo-1,4,5,6-tetrahydropyrrolo[3,4-*b*]pyrrol-2-yl)methylene)-2-oxoindolin-5-yl)methanesulfonamide (**10**)

The requisite *N*-(2-oxoindolin-5-yl)methanesulfonamide, for condensation with **3** to form target compound **10**, was prepared in 86% yield by a modified method of mesylation of 5-aminooxindole with methanesulfonyl chloride [33].

Yield of **10**: 54%, orange solids. Mp: 245–247 °C, UV λ_max_ (MeOH), nm (logɛ): 395 (4.43). IR (ATR), cm^−1^: 3539, 3260, 1681, 1586. ^1^H-NMR (400 MHz, acetic acid-*d*_4_) *δ*, ppm: 7.65 (s, 1H, –C=C**H**–), 7.61 (d, 1H, *J* = 2.0 Hz, Ar**H**), 7.16 (dd, 1H, *J* = 8.4, 2.0 Hz, Ar**H**), 6.99 (d, 1H, *J* = 8.4 Hz, Ar**H**), 4.58 (s, 2H, Ar–C**H**_**2**_), 3.98 (t, 2H, *J* = 6.0 Hz, –NC**H**_**2**_CH_2_-), 3.51 (t, 2H, *J* = 6.0 Hz, –NCH_2_C**H**_**2**_–), 3.89 (q, 4H, *J* = 7.2 Hz, –N(C**H**_**2**_CH_3_)_2_), 3.00 (s, 3H, –SO_2_C**H**_**3**_), 2.49 (s, 3H, Ar-C**H**_**3**_), 1.33 (t, 6H, *J* = 7.2 Hz, –N(CH_2_C**H**_**3**_)_2_). ^13^C-NMR (100 MHz, acetic acid-*d*_4_) *δ*, ppm: 171.5, 169.8, 149.7, 137.2, 133.6, 133.1, 127.3, 126.9, 126.3, 123.4, 121.0, 117.9, 115.1, 111.9, 52.0, 48.6, 48.2, 39.5, 10.0, 8.8. HRMS *m/z* (ESI): calcd., 472.2028 [M + H]^+^; found, 472.2013 [M + H]^+^.

#### (*Z*)-3-((5-(2-(Diethylamino)ethyl)-3-methyl-4-oxo-1,4,5,6-tetrahydropyrrolo[3,4-*b*]pyrrol-2-yl)methylene)-5-(trifluoromethyl)indolin-2-one (**11**)

Commercially available 5-trifluoromethyl-2-oxindoe was condensed with **7** to afford target compound **11** in a manner described above. Yield of **11**: 60%, orange solids. Mp: 212–215 °C, UV λ_max_ (MeOH), nm (logɛ): 415 (4.47). IR (ATR), cm^−1^: 3180, 1667, 1583. ^1^H-NMR (400 MHz, acetic acid-*d*_4_) *δ*, ppm: 7.71 (d, 1H, *J* = 2.0 Hz, Ar**H**), 7.57 (s, 1H, –C=C**H**–), 7.11 (dd, 1H, *J* = 8.4, 2.0 Hz, Ar**H**), 7.04 (d, 1H, *J* = 8.4 Hz, Ar**H**), 4.60 (s, 2H, Ar–C**H**_**2**_), 3.97 (t, 2H, *J* = 6.4 Hz, –NC**H**_**2**_CH_2_–), 3.49 (t, 2H, *J* = 6.4 Hz, –NCH_2_C**H**_**2**_–), 3.37 (q, 4H, *J* = 7.2 Hz, –N(C**H**_**2**_CH_3_)_2_), 2.48 (s, 3H, Ar–C**H**_**3**_), 1.31 (t, 6H, *J* = 7.2 Hz, –N(CH_2_C**H**_**3**_)_2_). ^13^C-NMR (100 MHz, acetic acid-*d*_4_) *δ*, ppm: 171.6, 169.7, 150.1, 145.8, 137.9, 133.6, 127.8, 127.7, 127.0, 123.1, 121.3, 117.3, 113.3, 112.0, 51.8, 48.6, 48.1, 39.5, 9.9, 8.7. HRMS *m/z* (ESI): calcd., 464.2241 [M + NH_4_]^+^; found, 464.1999 [M + NH_4_]^+^.

#### (*Z*)-3-((5-(2-(diethylamino)ethyl)-3-methyl-4-oxo-1,4,5,6-tetrahydropyrrolo[3,4-*b*]pyrrol-2-yl)methylene)-5-nitroindolin-2-one (**12**)

The requisite 5-nitrooxindole, for condensation with **3** to form target compound **12**, was prepared in 96% yield by a modified method of nitration of oxindole with HNO_3_/H_2_SO_4_ [33].

Yield of **12**: 62%, light yellow solids. Mp: 229–230 °C, UV λ_max_ (MeOH), nm (logɛ): 249 (4.62). IR (ATR), cm^−1^: 2971, 1671, 1553, 1551. ^1^H-NMR (400 MHz, acetic acid-*d*_4_) *δ*, ppm: 8.52 (d, 1H, *J* = 2.0 Hz, Ar**H**), 8.11 (dd, 1H, *J* = 8.4, 2.0 Hz, Ar**H**), 7.82 (s, 1H, –C=C**H**–), 7.11 (d, 1H, *J* = 8.4 Hz, Ar**H**), 4.60 (s, 2H, Ar–C**H**_**2**_), 3.99 (t, 2H, *J* = 6.0 Hz, –NC**H**_**2**_CH_2_–), 3.52 (t, 2H, *J* = 6.0 Hz, –NCH_2_C**H**_**2**_–), 3.40 (q, 4H, *J* = 7.2 Hz, –N(C**H**_**2**_CH_3_)_2_), 2.51 (s, 3H, Ar–C**H**_**3**_), 1.34 (t, 6H, *J* = 7.2 Hz, –N(CH_2_C**H**_**3**_)_2_). ^13^C-NMR (100 MHz, acetic acid-*d*_4_) *δ*, ppm: 171.6, 169.4, 150.5, 144.6, 143.8, 133.7, 128.7, 127.9, 127.1, 124.1, 121.5, 115.9, 115.2, 111.0, 51.8, 48.6, 48.1, 39.4, 10.0, 8.7. HRMS *m/z* (ESI): calcd., 424.1979 [M + H]^+^; found, 424.1992 [M + H]^+^.

#### (*Z*)-3-((5-(2-(diethylamino)ethyl)-3-methyl-4-oxo-1,4,5,6-tetrahydropyrrolo[3,4-*b*]pyrrol-2-yl)methylene)-5-methoxyindolin-2-one (**13**)

The requisite 5-methoxyoxindole, for condensation with **3** to form target compound **13**, was prepared in 78% yield by a modified method of Wolff-Kishner reduction of 5-methoxyisatin in the presence of N_2_H_4_ under basic conditions [34].

Yield of **13**: 59%, orange solids. Mp: 214–216 °C, UV λ_max_ (MeOH), nm (logɛ): 396 (4.69). IR (ATR), cm^−1^: 3028, 1672, 1577. ^1^H-NMR (400 MHz, acetic acid-*d*_4_) *δ*, ppm: 7.62 (s, 1H, –C=C**H**–), 7.25 (d, 1H, *J* = 2.0 Hz, Ar**H**), 6.91 (d, 1H, *J* = 8.4 Hz, Ar**H**), 6.79 (dd, 1H, *J* = 8.4, 2.0 Hz, Ar**H**), 4.59 (s, 2H, Ar–C**H**_**2**_), 3.98 (t, 2H, *J* = 6.0 Hz, –NC**H**_**2**_CH_2_–), 3.82 (s, 3H, –OC**H**_**3**_), 3.51 (t, 2H, *J* = 6.0 Hz, –NCH_2_C**H**_**2**_–), 3.39 (q, 4H, *J* = 7.2 Hz, –N(C**H**_**2**_CH_3_)_2_), 2.47 (s, 3H, Ar–C**H**_**3**_), 1.33 (t, 6H, *J* = 7.2 Hz, –N(CH_2_C**H**_**3**_)_2_). ^13^C-NMR (100 MHz, acetic acid-*d*_4_) *δ*, ppm: 171.5, 169.9, 157.1, 149.4, 133.5, 133.2, 127.3, 126.2, 125.5, 120.9, 118.9, 114.3, 112.0, 105.8, 55.6, 51.9, 49.2, 48.3, 39.5, 9.9, 8.8. HRMS *m/z* (ESI): calcd., 409.2234 [M + H]^+^; found, 409.2241 [M + H]^+^.

#### (*Z*)-3-((5-(2-(diethylamino)ethyl)-3-methyl-4-oxo-1,4,5,6-tetrahydropyrrolo[3,4-*b*]pyrrol-2-yl)methylene)-5-hydroxyindolin-2-one (**14**)

The requisite 5-hydroxyoxindole, for condensation with **3** to form target compound **14**, was prepared in 56% yield by a modified method of demethylation of 5-methoxyoxindole with a solution of hydrobromic acid in acetic acid [30].

Yield of **14**: 59%, orange solids. Mp: 240–242 °C, UV λ_max_ (MeOH), nm (logɛ): 359 (4.70). IR (ATR), cm^−1^: 3368, 3171, 1657, 1581. ^1^H-NMR (400 MHz, acetic acid -*d*_4_) *δ*, ppm: 7.50 (s, 1H, –C=C**H**–), 7.11 (d, 1H, *J* = 2.0 Hz, Ar**H**), 6.82 (d, 1H, *J* = 8.4 Hz, Ar**H**), 6.72 (dd, 1H, *J* = 8.4, 2.0 Hz, Ar**H**), 4.53 (s, 2H, Ar–C**H**_**2**_), 3.96 (t, 2H, *J* = 6.0 Hz, –NC**H**_**2**_CH_2_–), 3.50 (t, 2H, *J* = 6.0 Hz, –NCH_2_C**H**_**2**_–), 3.38 (q, 4H, *J* = 7.2 Hz, –N(C**H**_**2**_CH_3_)_2_), 2.45 (s, 3H, Ar–C**H**_**3**_), 1.33 (t, 6H, *J* = 7.2 Hz, –N(CH_2_C**H**_**3**_)_2_). ^13^C-NMR (100 MHz, acetic acid-*d*_4_) *δ*, ppm: 171.4, 169.9, 153.4, 149.3, 133.5, 132.6, 127.4, 125.9, 125.2, 120.7, 118.9, 115.4, 111.9, 106.9, 51.9, 48.6, 48.5, 48.2, 39.5, 9.9, 8.8. HRMS *m/z* (ESI): calcd., 395.2067 [M + H]^+^; found, 395.2078 [M + H]^+^.

#### (*Z*)-3-((5-(2-(diethylamino)ethyl)-3-methyl-4-oxo-1,4,5,6-tetrahydropyrrolo[3,4-*b*]pyrrol-2-yl)methylene)-5-mercaptoindolin-2-one (**15**)

The requisite 5-mercaptooxindole, for condensation with **3** to form target compound **15**, was prepared in 90% yield by treatment of 2-oxoindoline-5-sulfonyl chloride with triphenylphosphine [29].

Yield: 62%, orange solids. Mp: 257–260 °C, UV λ_max_ (MeOH), nm (logɛ): 394 (4.43). IR (ATR), cm^−1^: 3368, 3171, 1657, 1581. ^1^H-NMR (400 MHz, acetic acid-*d*_4_) *δ*, ppm: 7.66 (s, 1H, –C=C**H**–), 7.51–7.48 (m, 2H, Ar**H**), 7.06 (d, 1H, *J* = 8.4 Hz, Ar**H**), 4.67 (s, 2H, Ar–CH_2_), 4.00 (t, 2H, *J* = 5.6 Hz, –NC**H**_**2**_CH_2_–), 3.49 (t, 2H, *J* = 5.6 Hz, –NCH_2_C**H**_**2**_–), 3.39 (q, 4H, *J* = 7.0 Hz, –N(C**H**_**2**_CH_3_)_2_), 2.31 (s, 3H, Ar–C**H**_**3**_), 1.33 (t, 6H, *J* = 7.0 Hz, –N(CH_2_C**H**_**3**_)_2_). ^13^C-NMR (100 MHz, acetic acid-*d*_4_) *δ*, ppm: 171.3, 169.4, 149.8, 140.0, 133.5, 132.9, 131.8, 127.2, 127.0, 125.9, 123.9, 120.9, 117.2, 112.2, 51.8, 48.6, 48.1, 39.5, 9.9, 8.7. HRMS *m/z* (ESI): calcd., 410.1779 [M]^+^; found, 410.1771 [M]^+^.

### Biology

#### Cell culture

The HCT116 (human colon cancer cells, BCRC 60349) and Detroit 551 (human normal fibroblast cells, BCRC 60118) were maintained in DMEM (Gibco, Grand Island, NY, USA) containing 10% FBS (HyClone, Logan, UT, USA). NCI-H460 (BCRC 60373) and 786-O (BCRC 60243) cells were maintained in RPMI 1640 (Gibco, Grand Island, NY, USA) containing 10% FBS (HyClone, Logan, UT, USA). HUVEC (BCRC, H-UV001) was maintained in medium 199 (Sigma, St. Louis MO, USA) with 25 U/ml heparin (Sigma, St. Louis MO, USA), 30 μg/ml endothelial cell growth supplement (ECGS, Sigma, St. Louis MO, USA) containing 10% FBS (HyClone, Logan, UT, USA), and incubated at 37 °C in a 5% CO_2_ atmosphere.

#### Cell proliferation assay

The cells incubated as above were plated at a density of 2000 cells/well (cancer cells) [41, 44, 45] or 10,000 cells/well (Detroit 551) [46] on a 96-well plate for 24 h. Serial dilutions of indicated compounds were added and incubated for additional 72 h. At the end of the incubation, cell viability was determined by the 3-(4,5-dimethylthiazol-2-yl)-2,5-diphenyl tetrazolium bromide (MTT) assay. The MTT formazan crystals formed were dissolved in DMSO, and the absorbance at 570 nm was recorded using a microplate spectrophotometer (Thermo Fisher Scientific, Waltham, MA, USA) [46].

#### Acute cytotoxicity

The acute cytotoxicity effect of compounds **7** and **13**–**15**, and sunitinib was determined by Cell-Counting-Kit-8 (Dojindo, Rockville, MD, USA) assay on HCT116, NCI-460, 786-O, and Detroit 551 cells according to the manufacturer’s protocol. Cells were seeded at 5000 cells/well on a 96-well plate for 24 h. The indicated compounds in different concentrations (100 μl) were added to cells. After 6 h, old medium was aspirated, and the cells were washed three times with PBS. WST-8 (Dojindo, Rockville, MD, USA) (10 µl) was added to each well, and the absorbance of the plate was recorded at 450 nm on a microplate spectrophotometer (Thermo Fisher Scientific, Waltham, MA, USA).

#### Image cytometry

Cell cycle profiles of HCT116 cells were determined with an NC-3000 image cytometer (ChemoMetec, Allerod, Denmark) in accordance with manufacturer’s protocol. Briefly, cells were seeded at 200,000 cells/well on a 6-well plate for 24 h. Two ml of indicated compounds (5.0 μM for sunitinib and **7**, 3.0 μM for **13**–**15**) were added to cells. After incubation for 24 h, 100,000 cells were harvested and centrifuged at 400 g at room temperature for 5 min, washed once with PBS (50 μM), and resuspended in lysis buffer (50 μl) (ChemoMetec, Allerod, Denmark) containing 10 μg/ml of 2-(4-amidinophenyl)-1*H*-indole-6-carboxamidine (DAPI, ChemoMetec, Allerod, Denmark). The cells were incubated at 37 °C for 5 min and then stabilization buffer (50 μl) (ChemoMetec, Allerod, Denmark) was added to the mixture. The cellular fluorescence was measured with an NC-3000 image cytometer using NC-SlideA8 (ChemoMetec, Allerod, Denmark). The NC-3000 software (ChemoMetec, Allerod, Denmark) was used for image acquisition, image analysis and quantification, and data visualization.

#### In-vitro tube formation assay

The in vitro tube formation assay was assessed using ibidi μ-Slides (15-well, ibidi GmbH, Martinsried, Germany) in accordance with manufacturer’s protocol. Briefly, growth factor reduced Matrigel (10 μl) (Sigma, St. Louis MO) was added to the inner well of ibidi μ-Slides, and incubated at 37 °C for 1 h. HUVEC cells were harvested by centrifugation, and the cell suspension was adjusted to 200,000 cells/ml by 10 ng/ml VEGF contained growth medium (M199) with or without indicated compounds **7**, **13**, **14**, **15**, or sunitinib in different concentrations (1.0, 0.50 and 0.10 μM). 10,000 HUVEC cells in 50 μl of above growth medium was added to Matrigel (Sigma, St. Louis MO, USA) coated ibidi μ-Slides. After 6 h of incubation at 37 °C, the supernatant was discarded, and 50 μl of serum-free medium with diluted calcein AM (6.25 μg/ml) was added to above ibidi μ-Slides. After incubation in the dark at room temperature for 30 min, the μ-Slides were washed with PBS (50 μl) and fluorescence pictures were taken at 485 nm with a Leica DM1000 LED microscope (Leica Microsystems, Wetzlar, Hessen, Germany).

#### In-vitro kinase assay

The Reaction Biology Corporation (http://www.reactionbiology.com) HotSpot assay platform was used to determine the inhibitory activity of **7**, **13**, **14**, **15**, and sunitinib against VEGFR-2, PDGFRβ, and Aurora A, measured by quantifying the amount of ^33^P incorporated into the substrate in the presence of the test compound [47]. Briefly, specific kinase and substrate and required cofactors were prepared in reaction buffer. Test compounds were added to the reaction and after 20 min a mixture of ATP (Sigma, St. Louis MO, USA) and ^33^P ATP (Perkin Elmer, Waltham MA, USA) was added to make a final concentration of 10.0 μM. Reactions were stood at room temperature for 120 min, and then the reactions were spotted onto a P81 ion exchange filter paper (Whatman Inc., Piscataway, NJ, USA). Unbound phosphate was removed by extensive washing of filters in 0.1% phosphoric acid. Kinase activity data was reported as the percent remaining kinase activity in test compounds compared to the solvent control dimethyl sulfoxide (DMSO).

### Molecular modeling

Ligands-receptor docking calculation was carried out in accordance with the LibDock protocol. Briefly, receptor active site and ligands were characterized into polar and apolar hotspots. The ligand poses were placed into the receptor site in accordance with hotspots map. In this study CHARMm force field was used for energy minimization of the ligand molecules and ligand–receptor binding. The binding sphere was defined based on the protein data bank (PDB) definition. Conformations of ligands were generated by the BEST method.

### Statistical analysis

Statistical calculations were carried out with GraphPad Prism vision 5. Results are reported as the mean ± SD. Statistical significance was determined by the unpaired student’s *t* test.

## Additional file

**Additional file 1.** Additional figures.

## Abbreviations

EGF: epidermal growth factor
FGF: fibroblast growth factor
VEGF: vascular endothelial growth factor
PDGF: platelet-derived growth factor
RCC: renal cell carcinoma
GIST: gastrointestinal stromal tumor
pNET: pancreatic neuroendocrine tumor
PFS: progression free survival
SAR: Structure-activity relationship
EWG: electron-withdrawing group
HBD: hydrogen bond donating
NRP-1: neuropilin-1
SI: selectivity index
RTK: receptor tyrosine kinase
TMS: tetramethylsilane
LAH: lithium aluminum hydride
ATR: attenuated total reflection
TEA: triethylamine
MTT: 3-(4,5-dimethylthiazol-2-yl)-2,5-diphenyl tetrazolium bromide
DMSO: dimethyl sulfoxide
PDB: protein data bank
ESI: electrospray ionization
THF: tetrahydrofuran
