# Supplementary material for: Structural optimization and evaluation of novel 2-pyrrolidone-fused (2-oxoindolin-3-ylidene)methylpyrrole derivatives as potential VEGFR-2/PDGFRβ inhibitors
Source: Chem Cent J. 2017 Aug 1;11:72. doi: 10.1186/s13065-017-0301-5 (PMC5539068; doi:10.1186/s13065-017-0301-5)
Supplement: Supplementary file 1 — Additional file 1. Additional figures. [file 13065_2017_301_MOESM1_ESM.docx]

Additional file

**Structural Optimization and Evaluation of Novel 2-Pyrrolidone-fused (2-Oxoindolin-3-ylidene)methylpyrrole Derivatives as Potential VEGFR-2/PDGFR**β **Inhibitors**

Ting-Hsuan Yang^1^, Chun-I Lee^2^, Wen-Hsin Huang^2^, An-Rong Lee*^1,2^

^1^Graduate Institute of Medical Sciences, National Defense Medical Center, No. 161, Section 6, Mingchuan East Road, Taipei 11490, Taiwan; taffw9@gmail.com (T.-H. Y.)

^2^School of Pharmacy, National Defense Medical Center, No. 161, Section 6, Mingchuan East Road, Taipei 11490, Taiwan; acirlee@gmail.com (C.-I. L.), wenhsin1495@gmail.com (W.-H. H.)

***Corresponding author:**

An-Rong Lee

Tel: 886-2-8792-3100#18873

Email: [416806@gmail.com](mailto:416806@gmail.com)

NMR and IR spectra of **7** and **13**-**15**

**(*Z*)-*N*,*N*-Bis(2-chloroethyl)-3-((5-(2-(diethylamino)ethyl)-3-methyl-4-oxo-1,4,5,6-tetrahydropyrrolo[3,4-*b*]pyrrol-2-yl)methylene)-2-oxoindoline-5-sulfonamide (7)**

**
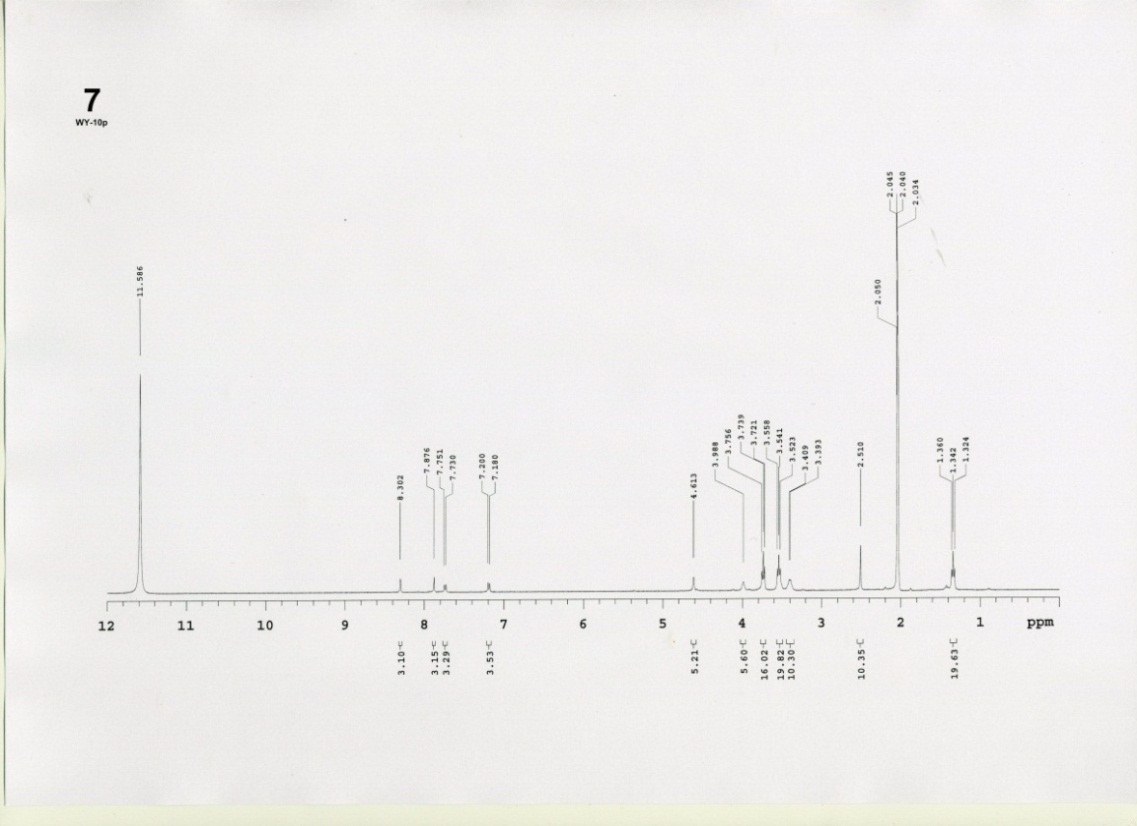
**

^1^H NMR of **7**


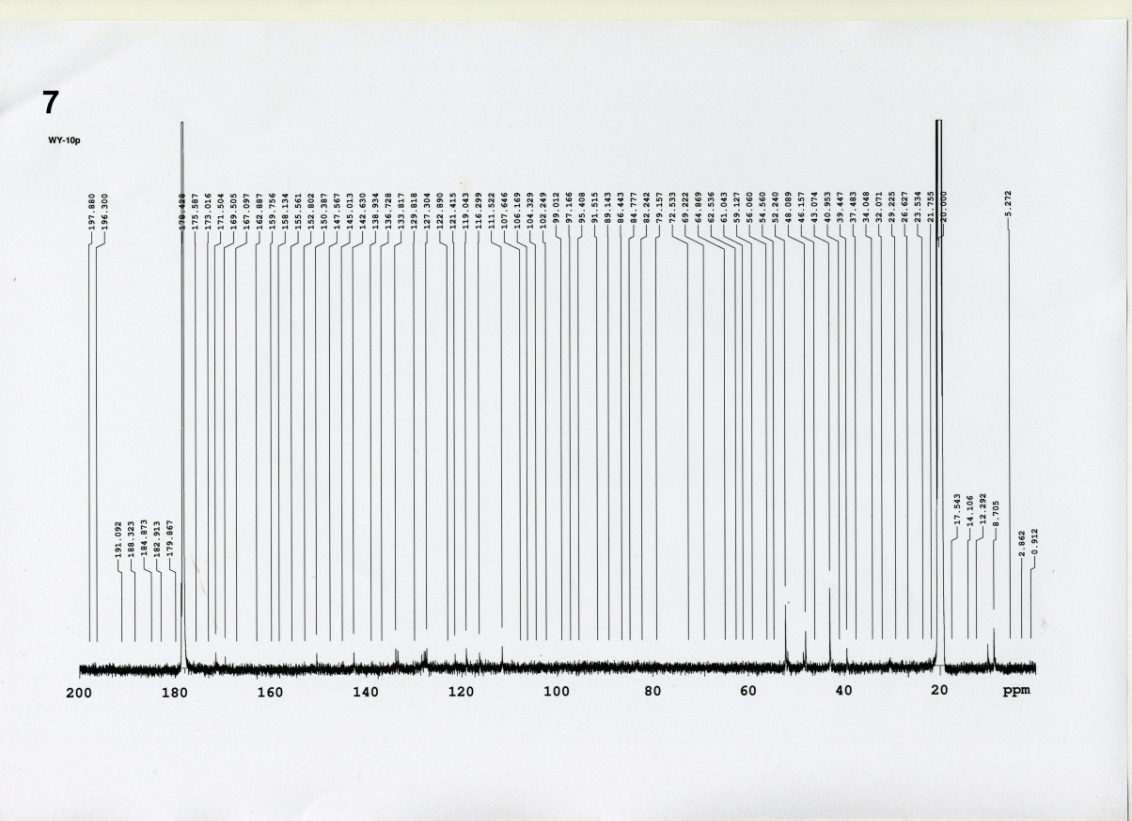


^13^C NMR of **7**


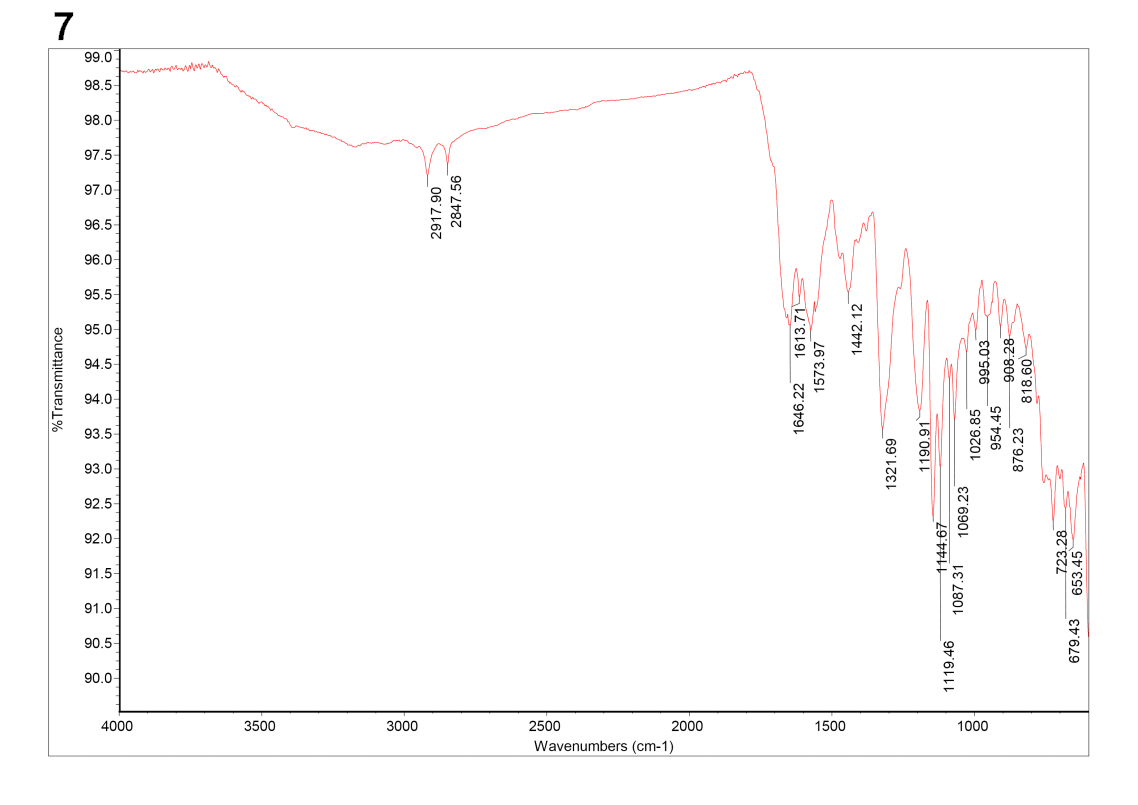


IR spectra of **7**

**(*Z*)-3-((5-(2-(Diethylamino)ethyl)-3-methyl-4-oxo-1,4,5,6-tetrahydropyrrolo[3,4-*b*]pyrrol-2-yl)methylene)-5-methoxyindolin-2-one (13)**
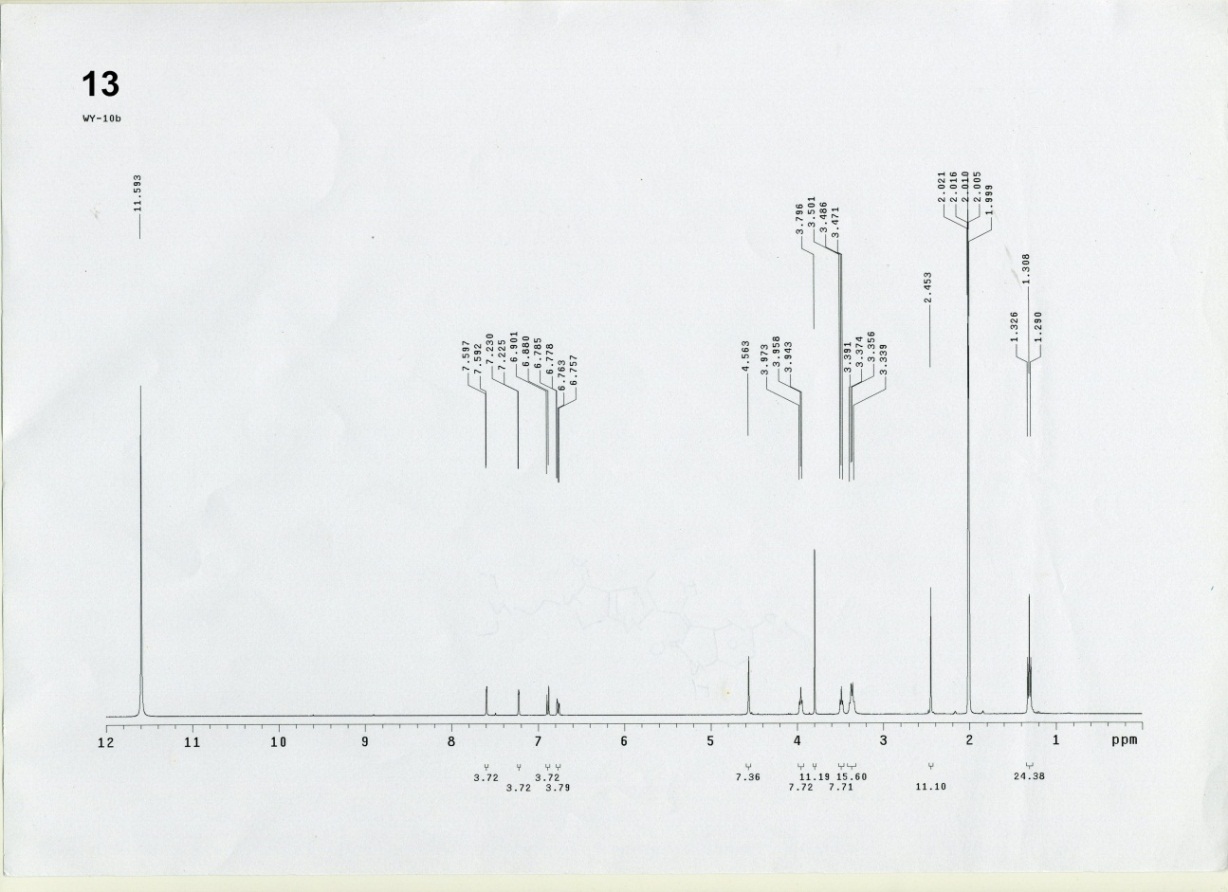


^1^H NMR of **13**


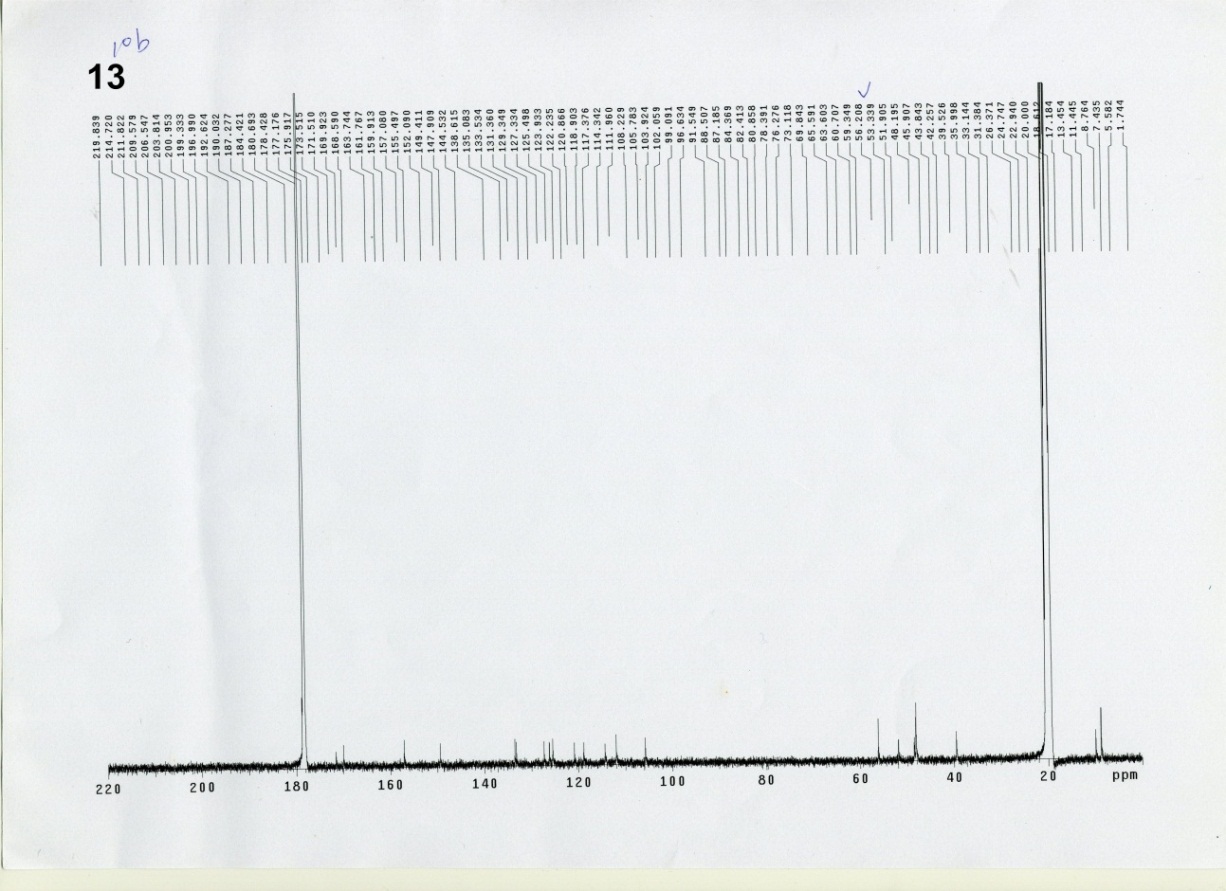


^13^C NMR of **13**


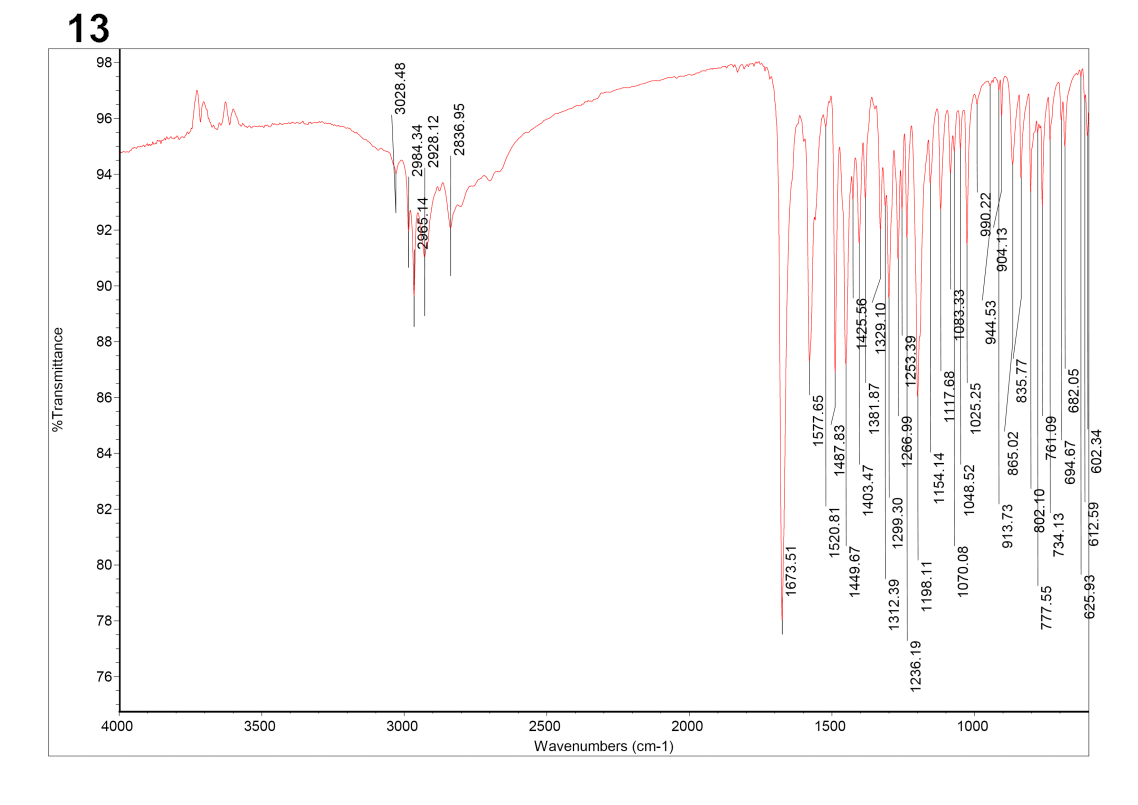


IR spectra of **13**

**(*Z*)-3-((5-(2-(Diethylamino)ethyl)-3-methyl-4-oxo-1,4,5,6-tetrahydropyrrolo[3,4-*b*]pyrrol-2-yl)methylene)-5-hydroxyindolin-2-one (14)**


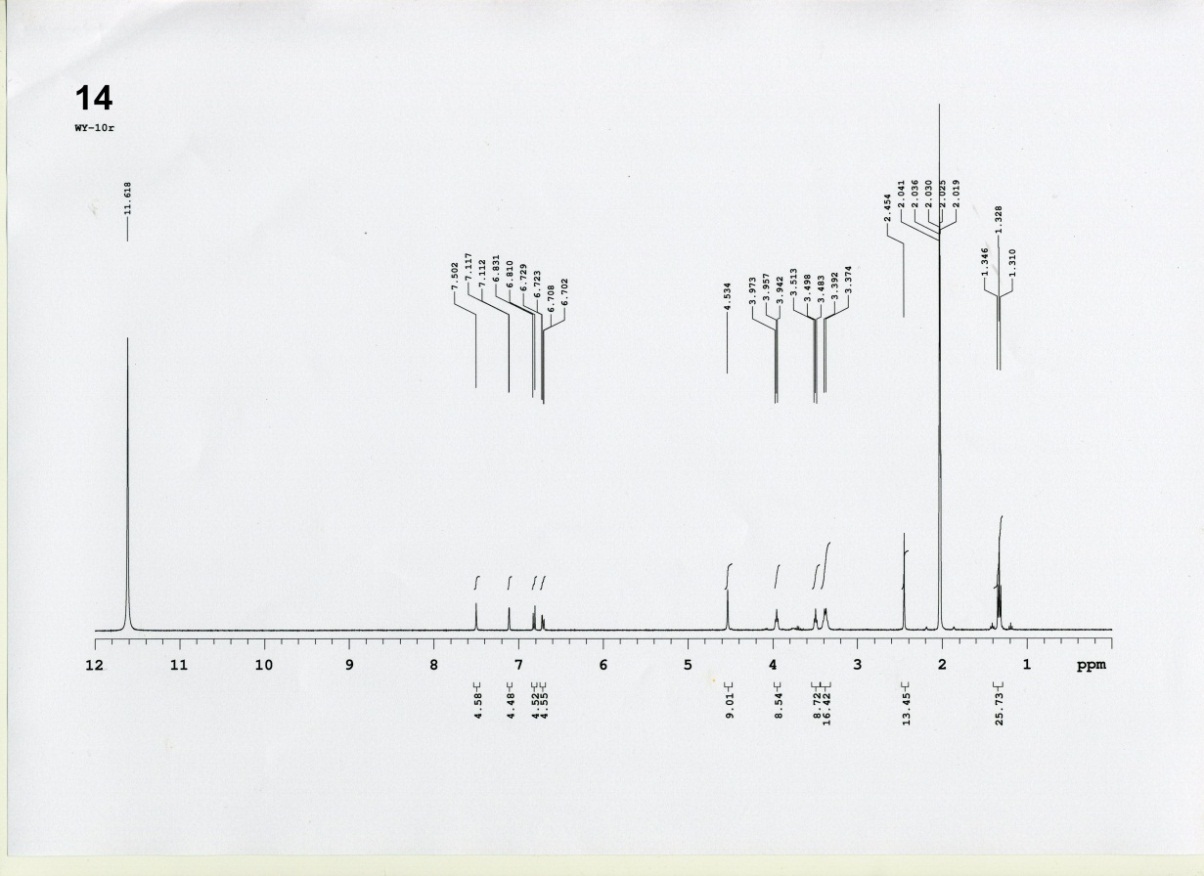


^1^H NMR of **14**


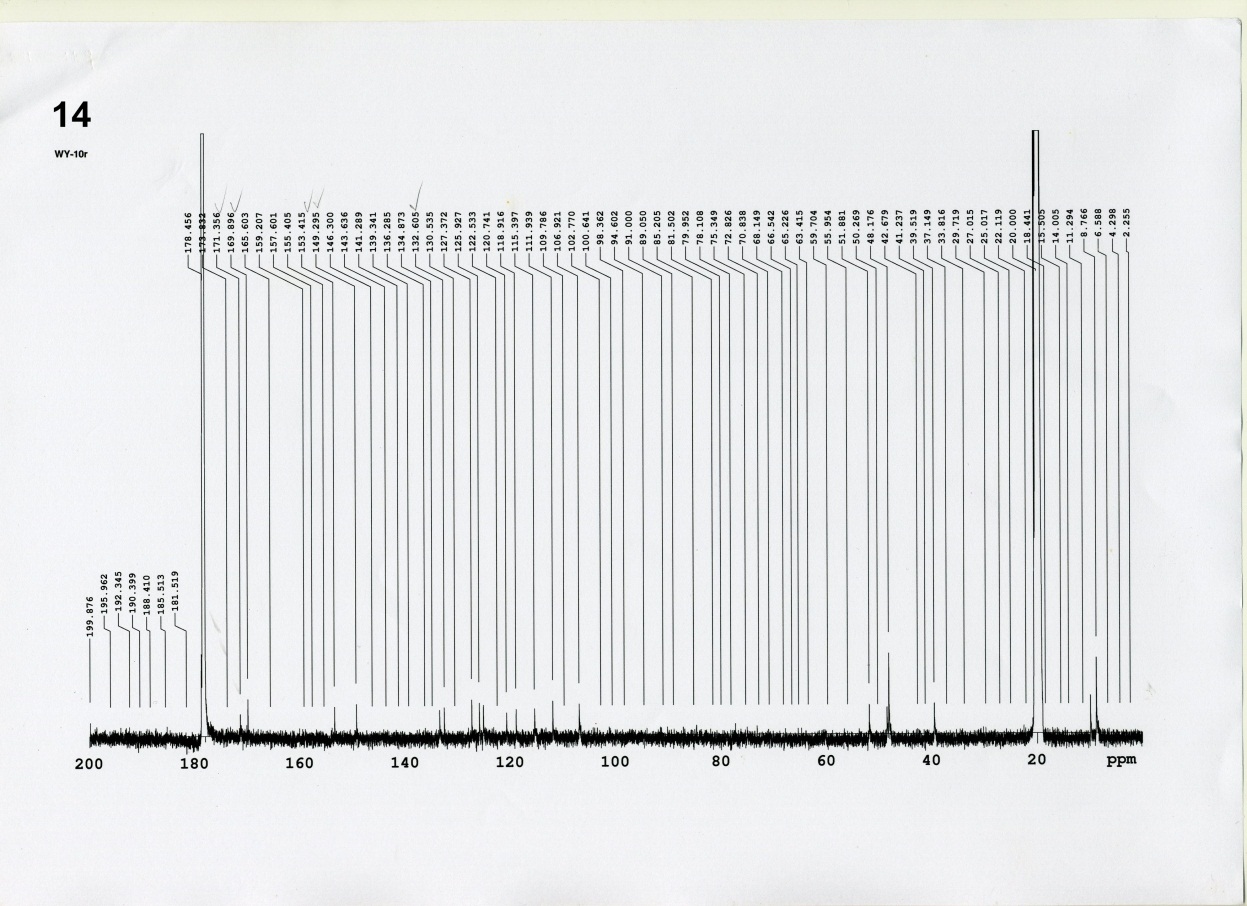


^13^C NMR of **14**


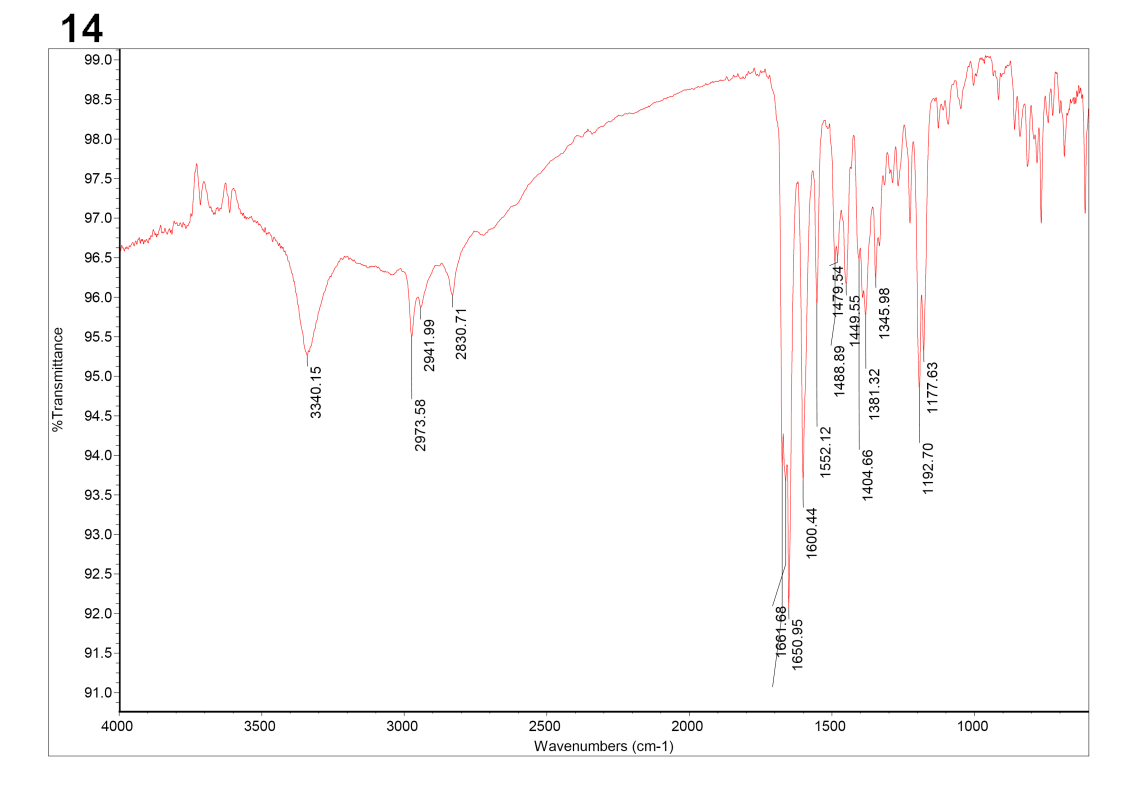


IR spectra of **14**

**(*Z*)-3-((5-(2-(Diethylamino)ethyl)-3-methyl-4-oxo-1,4,5,6-tetrahydropyrrolo[3,4-*b*]pyrrol-2-yl)methylene)-5-mercaptoindolin-2-one (15)**


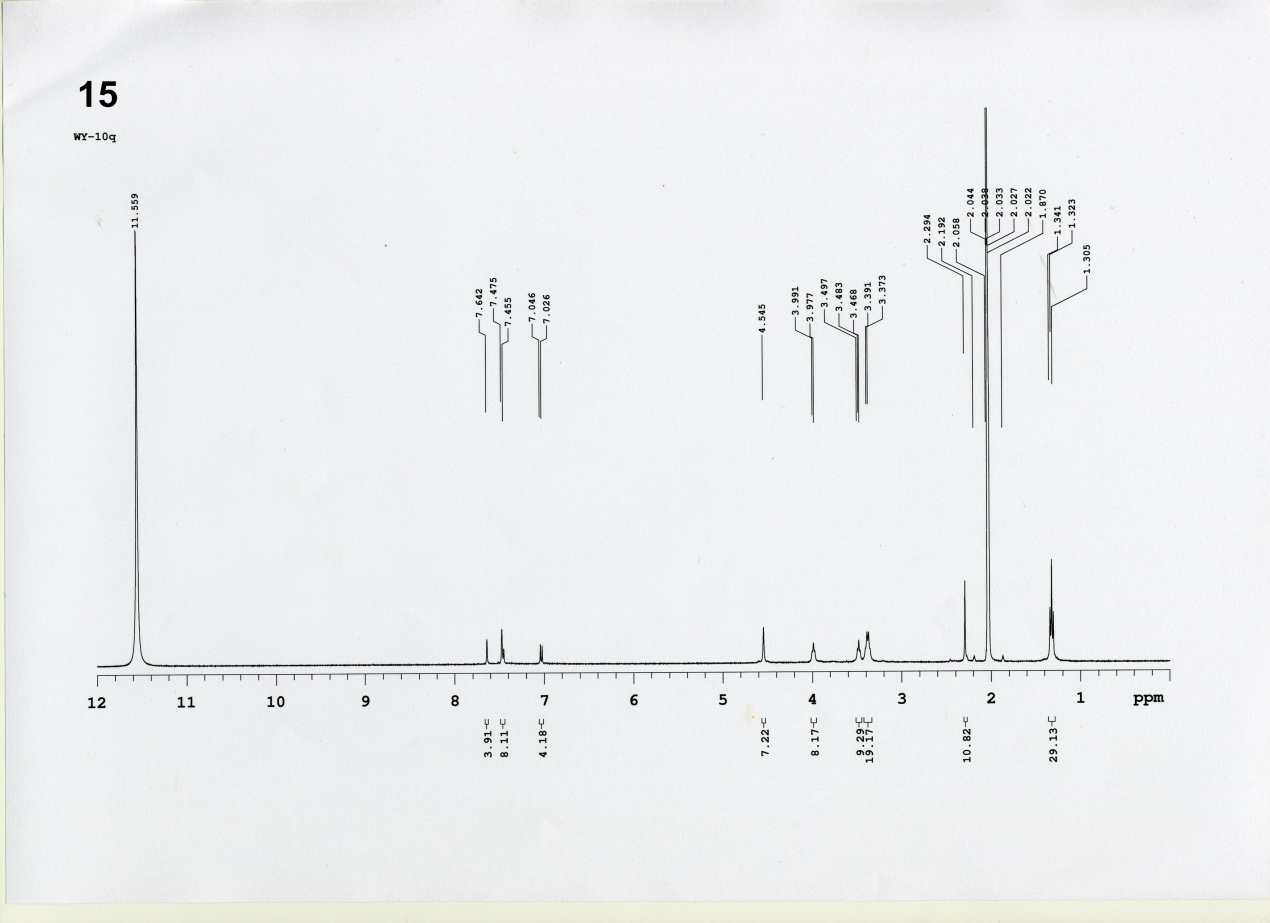
^1^H NMR of **15**


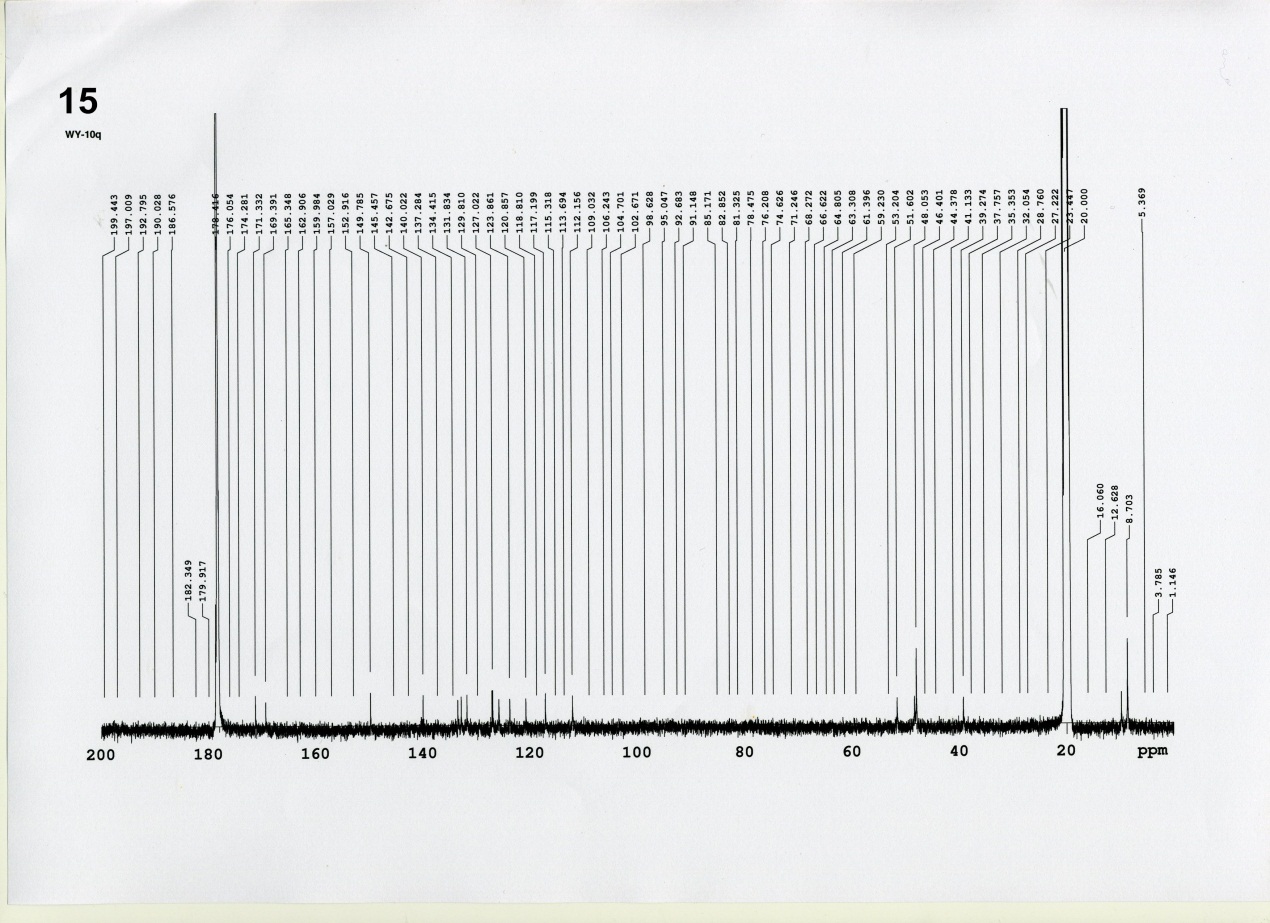


^13^C NMR of **15**


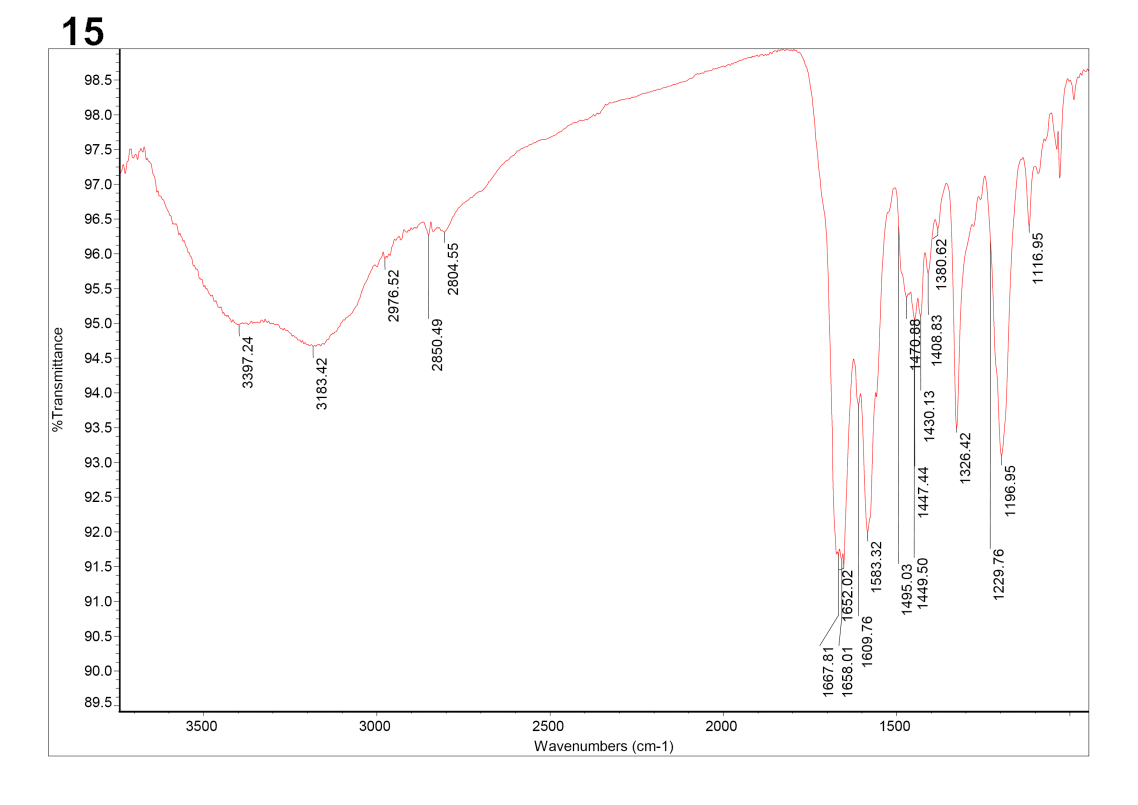


IR spectra of **15**
